# Machine learning and multi-omics data reveal driver gene-based molecular subtypes in hepatocellular carcinoma for precision treatment

**DOI:** 10.1371/journal.pcbi.1012113

**Published:** 2024-05-10

**Authors:** Meng Wang, Xinyue Yan, Yanan Dong, Xiaoqin Li, Bin Gao

**Affiliations:** Faculty of Environment and Life of Beijing University of Technology, Beijing, China; University of Southern California, UNITED STATES

## Abstract

The heterogeneity of Hepatocellular Carcinoma (HCC) poses a barrier to effective treatment. Stratifying highly heterogeneous HCC into molecular subtypes with similar features is crucial for personalized anti-tumor therapies. Although driver genes play pivotal roles in cancer progression, their potential in HCC subtyping has been largely overlooked. This study aims to utilize driver genes to construct HCC subtype models and unravel their molecular mechanisms. Utilizing a novel computational framework, we expanded the initially identified 96 driver genes to 1192 based on mutational aspects and an additional 233 considering driver dysregulation. These genes were subsequently employed as stratification markers for further analyses. A novel multi-omics subtype classification algorithm was developed, leveraging mutation and expression data of the identified stratification genes. This algorithm successfully categorized HCC into two distinct subtypes, CLASS A and CLASS B, demonstrating significant differences in survival outcomes. Integrating multi-omics and single-cell data unveiled substantial distinctions between these subtypes regarding transcriptomics, mutations, copy number variations, and epigenomics. Moreover, our prognostic model exhibited excellent predictive performance in training and external validation cohorts. Finally, a 10-gene classification model for these subtypes identified *TTK* as a promising therapeutic target with robust classification capabilities. This comprehensive study provides a novel perspective on HCC stratification, offering crucial insights for a deeper understanding of its pathogenesis and the development of promising treatment strategies.

## Author summary

Dividing highly heterogeneous HCC into molecular subtypes with similar characteristics is crucial for personalized anti-tumor therapies. Although driver genes play pivotal roles in cancer progression, their potential in HCC subtyping has been largely overlooked. In this work, we developed a multi-omics network-based stratification algorithm that utilizes patient mutation data and requires smaller computational resources for subtype assignment. Through this algorithm, we categorized HCC into two subtypes, CLASS A and CLASS B. Using multi-omics and single-cell data, we identified differences between these subtypes in gene expression, methylation, immune infiltration, and other aspects. Beyond subtype characterization, our study established a robust clinical prediction model (https://mike-wang-bjut.shinyapps.io/DynNomapp_HCC_Sutypes/) incorporating subtype information and typical clinical features, enabling precise survival predictions. Finally, we developed a high-performing machine learning classifier for our subtype. Analyzing this classification model and reviewing previous experimental papers, we identified *TTK* as a potential diagnostic marker and therapeutic target specific to our subtypes. In conclusion, our research offers a novel perspective on HCC stratification, which is crucial for a deeper understanding of its pathogenesis and developing promising treatment strategies.

## Introduction

Hepatocellular carcinoma (HCC) is recognized as the most prevalent primary liver malignancy, ranking as the third leading cause of cancer-related deaths globally and experiencing a notable increase in incidence [1,2]. The molecular and pathological heterogeneity of HCC presents a formidable obstacle to developing personalized therapeutic approaches [3]. Therefore, using key features to classify different HCC patients into relatively homogeneous subtypes is clinically essential.

Recent advances in high-throughput sequencing technologies have facilitated the comprehensive profiling of patient dysfunctions across multiple biological systems. Through various omics techniques, potential oncogenic factors can be discerned [4]. Utilizing big data in molecular subtyping of HCC has become increasingly feasible [5]. For instance, Zhang et al. employed mass cytometry data to categorize HCC into three subtypes, each exhibiting diverse immune activities [6]. Poirion et al. developed the DeepProg deep learning method, which integrates RNA, DNA methylation, and miRNA data to classify HCC into two subtypes with distinct survival differences and biological profiles [7].

In tumors, driver genes are often causally related to tumor progression. Compiling a complete list of driver genes is crucial for oncology diagnosis and drug development [8]. We have identified recurrently altered driver genes in HCC, and some of these genes have been suggested to be associated with specific molecular subtypes [9]. However, no systematic subtyping studies of HCC using driver genes exist to our knowledge.

Gene families, representing a cluster of genes with shared ancestry and similar biochemical functions, present an opportunity to identify rare carcinogenic mutations [10–12]. In this study, we utilized gene families to expand the list of driver genes, culminating in creating two distinct molecular subtypes for HCC. By integrating multi-omics data and single-cell information, we explored the unique characteristics defining these subtypes, spanning transcriptomics, genomics, epigenomics, immune infiltration, and tumor stem cell activity. Additionally, we developed an interactive prognostic model website for the two subtypes, empowering users to effortlessly generate personalized survival predictions based on critical patient information, including age, stage, virus infection status, and subtype classification. Finally, we established a 10-gene classification model for these subtypes and singled out *TTK* as a promising therapeutic target with strong classification capabilities. In summary, our study provides a fresh perspective on the construction of HCC subtypes and offers promising avenues for future therapeutic strategies.

## Result

### Obtaining stratification genes from driver genes and their family members

To ensure a comprehensive and high-confidence selection of driver genes, we gathered HCC driver gene lists from three distinct studies. Bailey et al. [13] employed diverse driver gene discovery algorithms and conducted meticulous manual curation to construct their driver gene list. Martínez-Jiménez et al. [14] extended the scope by analyzing a larger sample size and adopting a more comprehensive approach to exploring driver genes. Meanwhile, Fujimoto et al. [15] concentrated on HCC, providing valuable insights into the specific driver mechanisms of HCC. By amalgamating the findings of these three studies, we curated a list of 96 HCC driver genes for our subsequent analyses. Furthermore, we enriched this selection by including their corresponding family members, which were sourced from InterPro (https://www.ebi.ac.uk/interpro/), UniProtKB (https://www.uniprot.org/), as well as several other references [16–21]. (S1 Table).

**Fig 1:**
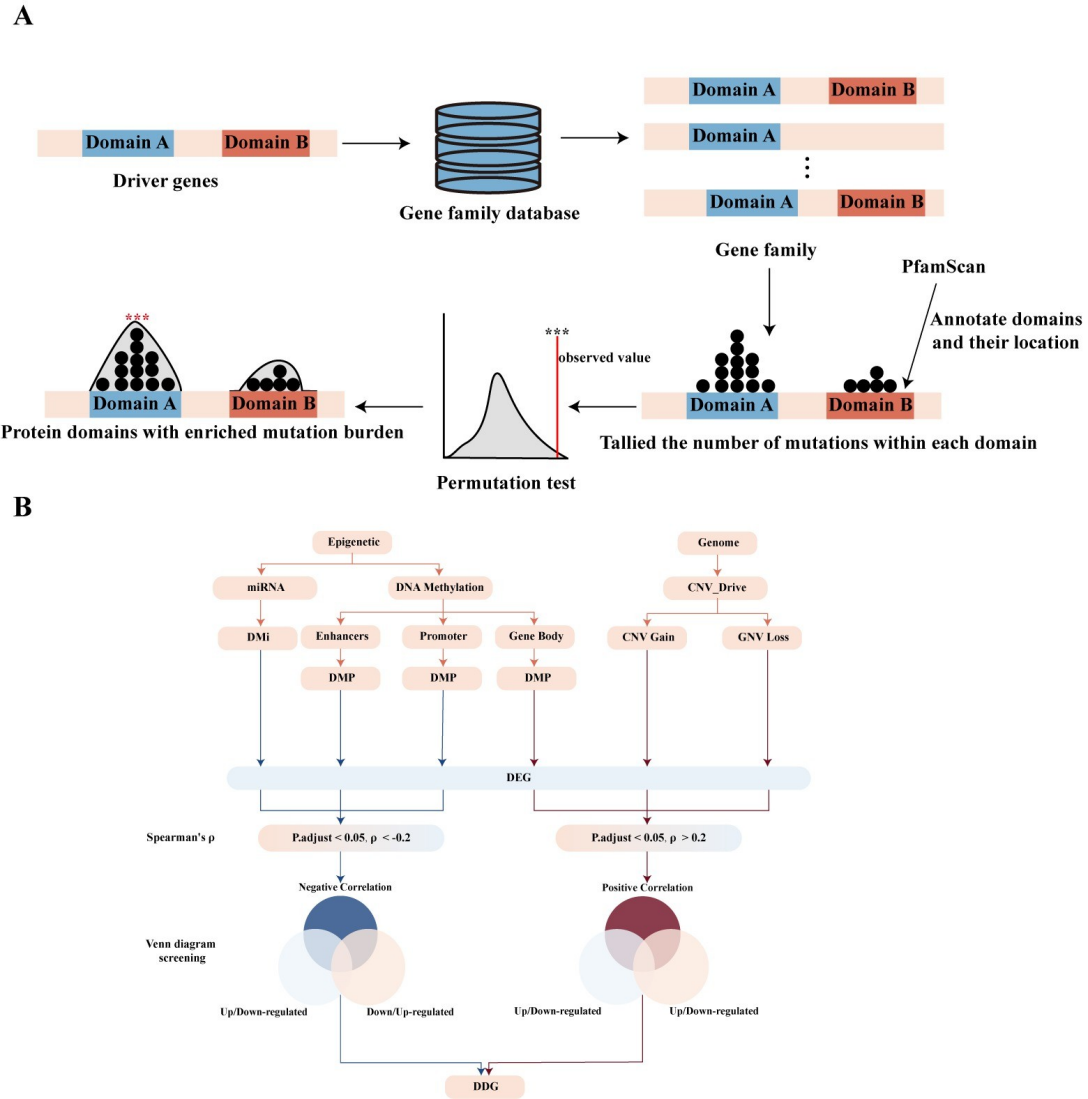
illustrates the process of obtaining stratification genes. (A) The workflow for protein domains with significant mutation burden (B) illustrates the process of defining DDGs.

To identify protein domains with significant mutation burden, we first annotated the domains for each gene using the PfamScan (https://www.ebi.ac.uk/Tools/pfa/pfamscan/) and excluded those with an e-value greater than 1e-5. Additionally, considering the potential role of the Degron region in transcription factors for cancer growth [22], we included Degron as a protein domain based on Degpred predictions [23]. Through permutation testing, we identified 75 protein domains with significant mutation burden (Fig 1A). Focusing on protein domains with higher entropy values, which may indicate novel oncogenic alterations, we identified 1192 stratification genes mutated in domains with entropy values greater than 0.5 (S2 and S3 Tables).

The progression of HCC involves genetic mutations, epigenetic variations, and dysregulated gene expression [24–26]. Many well-known HCC driver gene alterations are associated with an epigenetic variation or copy number variation (CNV). To complete the stratification genes, we conducted differential analysis between normal and tumor tissues, identifying 244 differentially expressed genes within gene families. Then, we integrated other omics data to define 223 driver-dysregulated genes (DDGs) (Fig 1B and S3 Table).

### Driver gene-related HCC Subtypes

**Fig 2:**
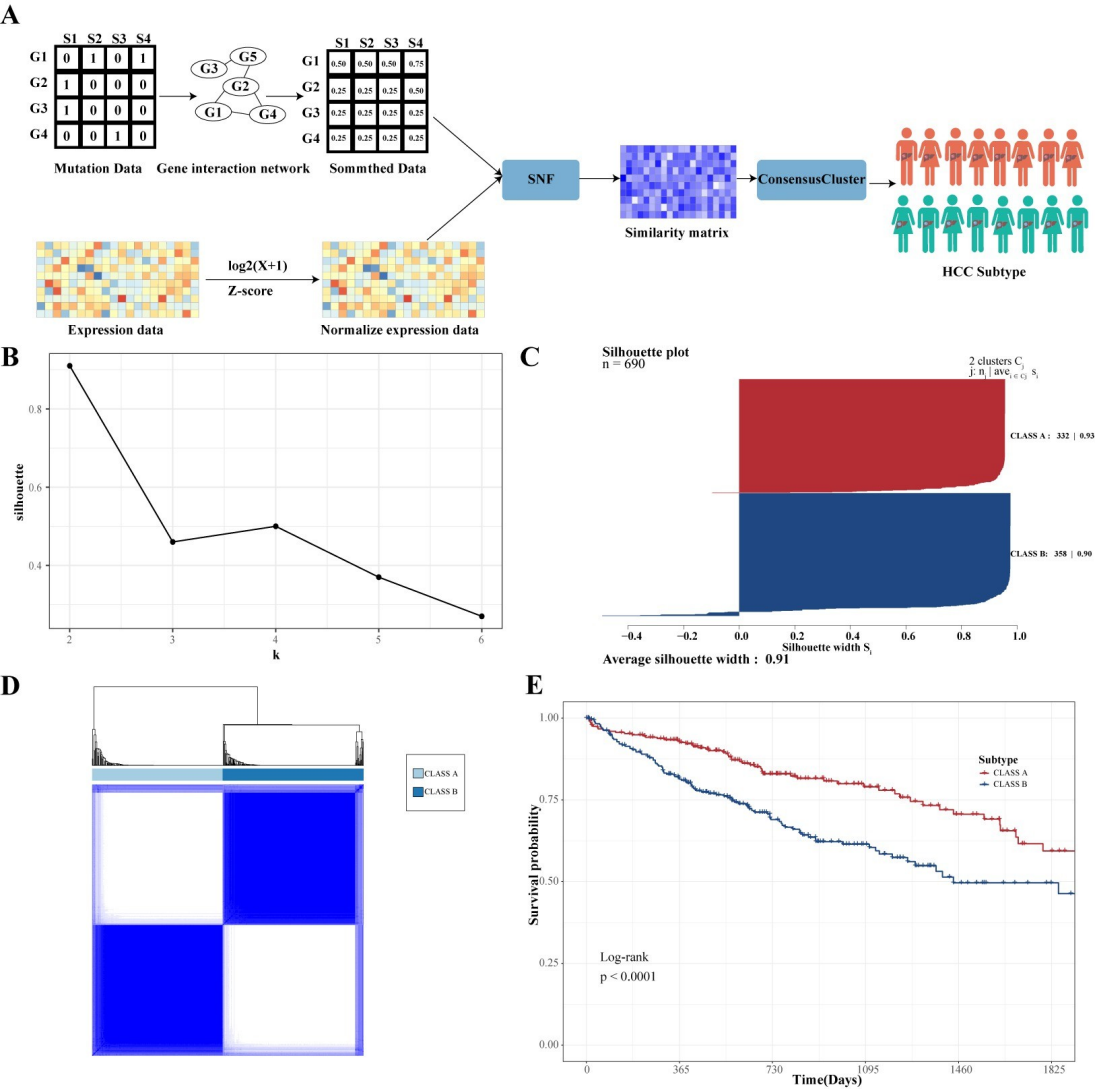
Identification of driver gene-related subtypes. (A) Flowchart depicting the process of the subtype classification algorithm; (B) Silhouette coefficients for different values of k; (C) Silhouette plot specifically for k = 2; (D) Heatmap of the consensus matrix defining the two subtypes; (E) Five-year survival curves for the two subtypes, with CLASS A represented in red and CLASS B represented in blue.

We stratified HCC into distinct subtypes using mutation data from stratification genes and DDGs expression data. Firstly, we smoothed the patient mutation matrix using the Network-based stratification (NBS) algorithm [27]. Next, we integrated the smoothed mutation data with DDGs expression data using the Similarity network fusion (SNF) algorithm to construct a similarity matrix among samples [28]. The amalgamation of SNF with a consensus cluster facilitated patient subtype assignment, yielding more robust and desirable clustering outcomes (Fig 2A, Table 1). The combination of smoothed mutation data and DDG expression data proved effective in capturing information and enhancing the clustering analysis.

**Table 1:** Comparison of different clustering methods.

Method	Mutation data	Expression Data	Network	Num. subtypes	Sil	p-value
CC	None	DDG expression data	None	2	0.72	1.687729e-08
SNF+CC	None	DDG expression data	None	2	0.83	0.00012
SNF+CC	Binary matrix	DDG expression data	None	2	0.82	0.00588
SNF+CC	Smoothed data	DDG expression data	String	2	0.91	8.52e-05
**SNF+CC**	**Smoothed data**	**DDG expression data**	**Human Netv3**	**2**	**0.91**	**7.88e-05**
SNF+CC	Driver gene Smoothed data	DDG expression data	Human Netv3	2	0.75	0.082

SNF: Similarity network fusion, CC: ConsensusCluster, Sil: Silhouette Coefficient

Choosing the suitable gene interaction network is crucial for the NBS algorithm’s smoothing effect. We compared two commonly used networks, String (Requested score = medium confidence, FDR stringency = medium) and HumanNetv3 (top 10% confidence) [29]. We found that HumanNetv3 performed better in stratification based on silhouette coefficients and p-values (Table 1). Additionally, we examined the stratification outcomes by utilizing only driver genes versus incorporating gene family members for HCC. The results demonstrated that the latter approach exhibited superior performance in both clustering stability and survival differences, as reflected in silhouette coefficients and p-values (Table 1).

To determine the optimal cluster count, we computed silhouette coefficients for various cluster numbers (k), revealing that k = 2 was the most suitable parameter for delineating HCC subtypes (Fig 2B and 2C). We designated these subtypes as CLASS A and CLASS B (Fig 2D), where patients with CLASS B had worse prognostic outcomes (median OS time 17 months vs 20.55 months) (Fig 2E). Subsequent validation of our method on the TCGA_LIHC cohort further confirmed significant survival differences between the two subtypes (median OS time 15.97 months vs 22.42 months) (S1 Fig). This demonstrates that integrating smoothed mutation data with gene expression data effectively classifies HCC subtypes.

Our identified HCC subtypes significantly correlated with independently studied subtypes [30–33] (chi-square test, see S4 and S5 Tables, S2 Fig). We constructed Cox regression models based on these subtypes. We assessed predictive performance using the C-index and Integrated Brier Score (IBS) to evaluate the association of different subtypes with clinical prognosis. The results demonstrate that our constructed subtypes excel in C-index, reaching 0.611 (95% CI = 0.587–0.635), significantly higher than most other models (S3 Fig). Simultaneously, the IBS is 0.183, lower than other models (Hoshida: 0.195, Bidkhori: 0.205, Benfeitas: 0.194, TCGA: 0.180). This indicates that our subtypes exhibit higher consistency and accuracy in predicting patient survival than others.

### Biological properties of different subtypes

**Fig 3:**
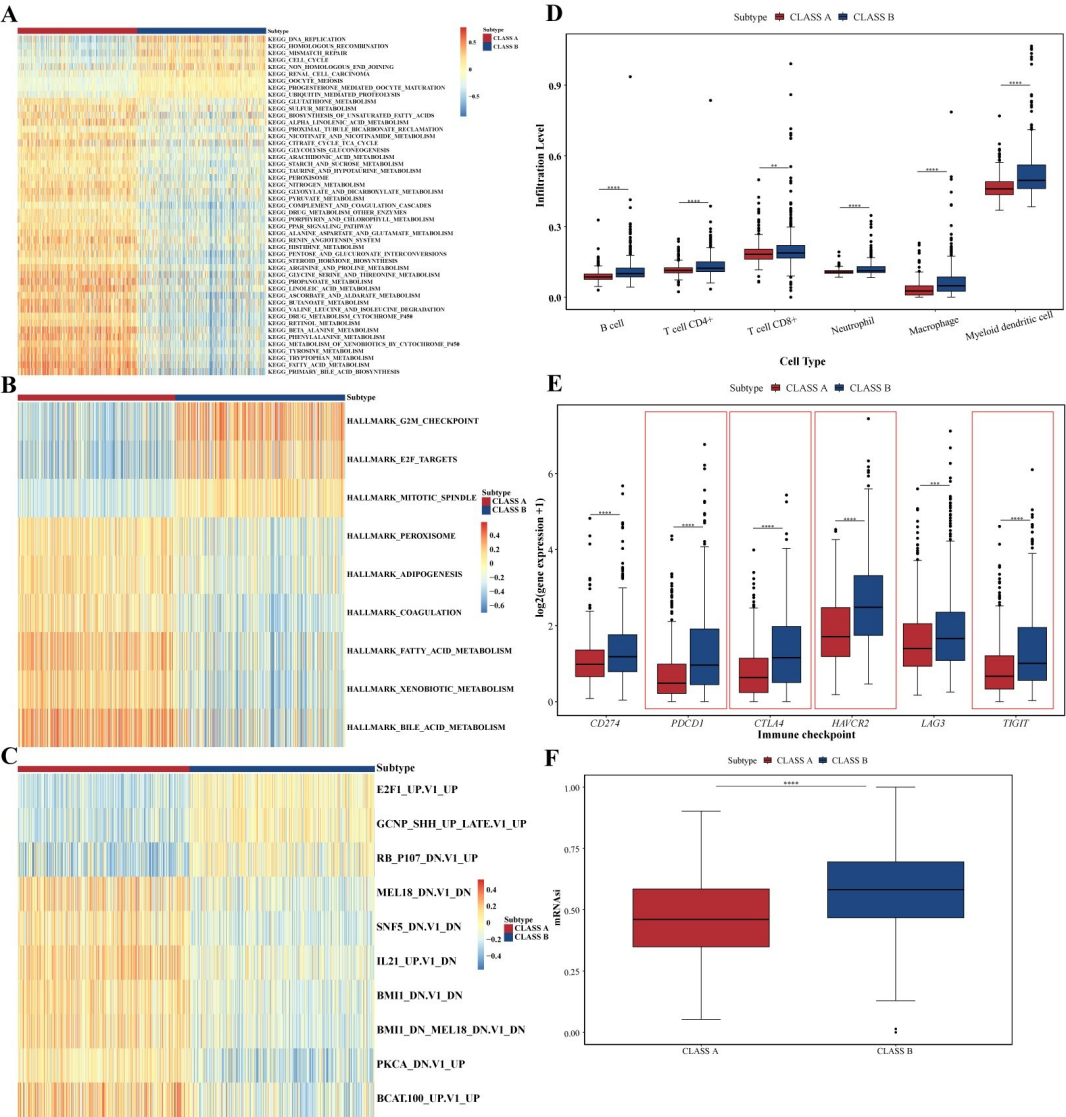
Different biological properties of the two subtypes. (A-C) Differential analysis of KEGG, Hallmark and oncogenic signature pathways between these two subtypes. (D) The immune cell abundance in these two subtypes using TIMER, with statistical significance assessed by the Mann-Whitney U test. (E) Box plots depicting the expression levels of six immune checkpoint genes in these two subtypes, with red boxes indicating significantly differentially expressed genes (p.adjust < 0.05, |log2FC| > 1). (F) Box plot showing the mRNA stemness scores (mRNAsi) of the two subtypes.

To explore distinct biological properties, we performed gene set variation analysis (GSVA) on KEGG, Reactome, Hallmark, and oncogenic signature gene sets for the two subtypes. The results revealed high consistency in enrichment patterns across these gene sets for both subtypes (Fig 3A–3C and S6 Table). CLASS A showed higher GSVA scores in metabolic pathways such as oxidative phosphorylation, organic acid metabolism, fatty acid metabolism, and glycolysis. In contrast, CLASS B displayed a heightened proliferative profile, enriched in pathways associated with mitosis, cell cycle, DNA replication, and cell cycle checkpoint. Additionally, CLASS B had upregulated activity in pathways related to histone methylation, DNA methylation, and inflammatory signaling (S6 Table).

Next, we analyzed immune cell composition using TIMER [34] and xCell algorithms [35], revealing a higher abundance of immune cells, including T cells, B cells, and macrophages, in CLASS B than CLASS A. CLASS B had a higher stromal score. In contrast, CLASS A had a higher microenvironmental score (Figs 3D, S4A and S4B).

We also examined the expression of six common immune checkpoints [36,37], finding higher expression levels in CLASS B than in CLASS A (Fig 3E). Specifically, PD-1 (*PDCD1*), CTLA-4 (*CTLA4*), TIM-3 (*HAVCR2*), and *TIGIT* were differentially expressed in CLASS B, suggesting potential responsiveness to immune checkpoint inhibitors.

Finally, we used Malta et al.’s machine learning [38] algorithm to examine the stemness features of two subtypes. The results showed that CLASS B exhibited stronger stemness features, indicating a higher potential for invasion and metastasis (Fig 3F). This alteration may be associated with the high expression of *KRT19*, a marker for biliary/hepatic progenitor cells, in CLASS B (S5 Fig) [39].

### Multi-omics properties of subtypes

**Fig 4:**
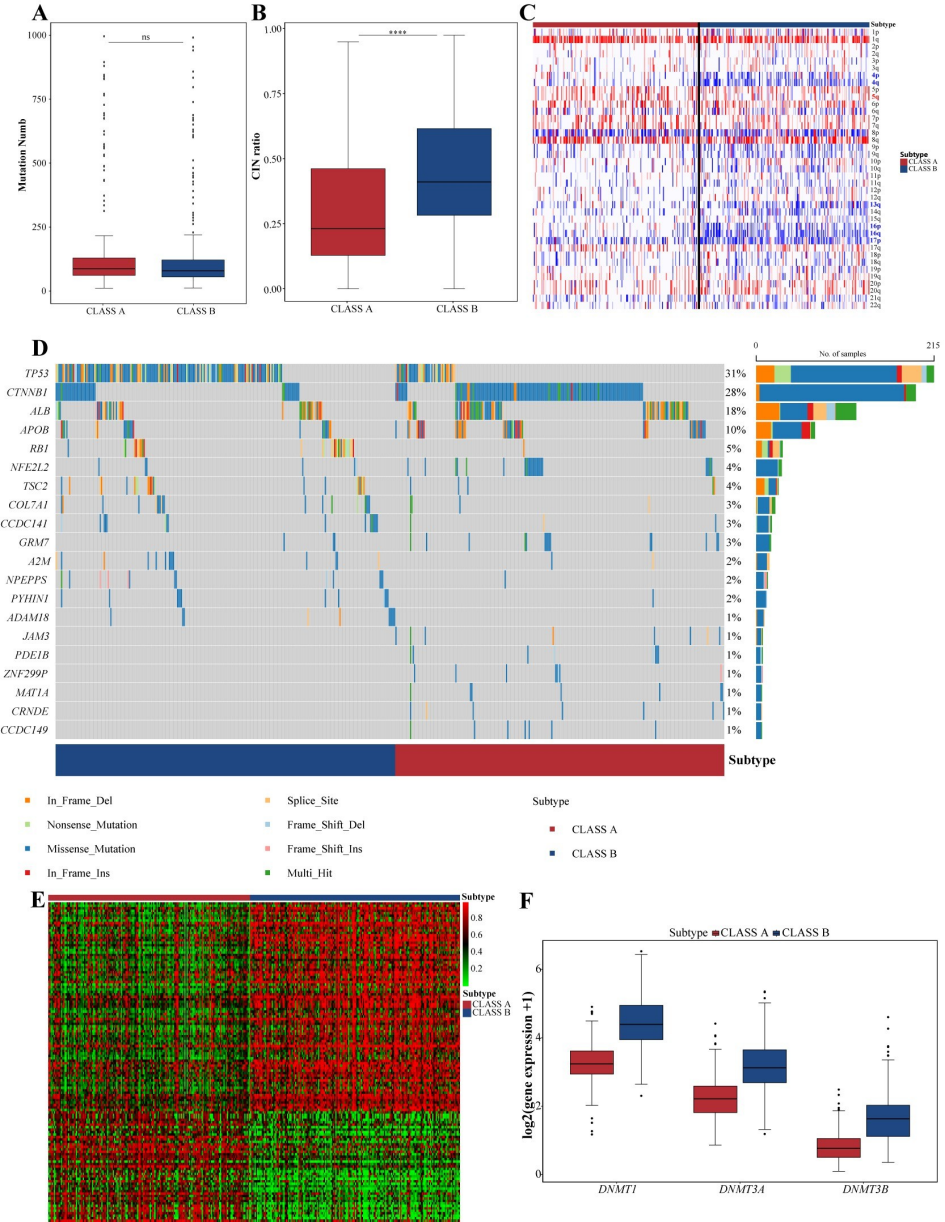
Distinct multi-omics features of the two subtypes. (A) Box plot showing the number of nonsynonymous mutations in each subtype. (B) Box plot displaying the CIN ratio in each subtype. (C) Heatmap illustrating CNVs of the 22 autosomes in both subtypes, with red and blue indicating copy number amplifications and deletions, respectively. (D) Oncoplot presents the top 20 significantly mutated genes in the subtypes based on the p-values. (E) Identifying of subtype-specific methylation probes in the two groups using the R package ChAMP, with a threshold of p.adjust < 0.05 and |Δβ| > 0.2. (F) Expression profiles of DNA methyltransferase family members DNMT1, DNMT3A, and DNMT3B in these two subtypes.

Despite no significant difference in mutation count and TMB values between the subtypes (Figs 4A and S6), CLASS B showed higher chromosomal instability (Fig 4B). Specifically, CLASS B displayed deletions in 4p, 4q, 13q, 16p, 16q, and 17p, while CLASS A predominantly manifested amplifications in 5q (Fig 4C).

Using the chi-square test, we identified significantly mutated genes in these subtypes (Fig 4D). *TP53* and *CTNNB1*, well-known HCC driver genes, had different distributions between subtypes. Consistent with previous research, *TP53* was mainly found in CLASS B, associated with a poorer prognosis, while *CTNNB1* was mainly associated with CLASS A, linked to a better prognosis [40,41]. Additionally, *RB1* mutations were predominantly enriched in CLASS B, possibly contributing to its high proliferative profile (S6 Table).

By analyzing TCGA data, we found significant hypermethylation patterns in CLASS B (Fig 4E). This might link to the high expression of DNA methyltransferase family members (*DNMT1*, *DNMT3A*, and *DNMT3B*) in this subtype (Fig 4F).

### Machine learning-based diagnostic models for HCC subtypes and identify potential therapeutic targets

**Fig 5:**
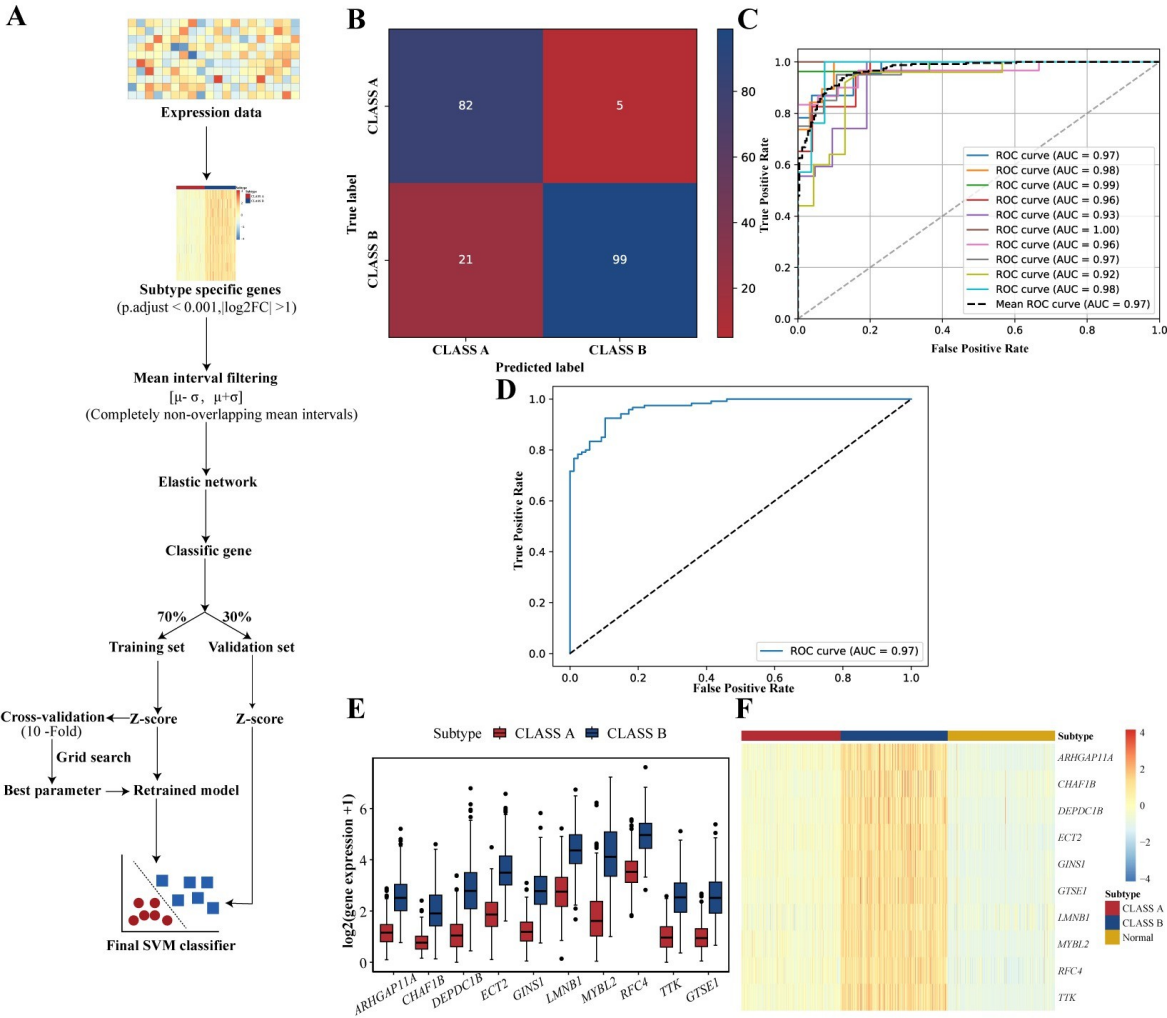
Machine learning classifier for HCC subtypes. (A) Workflow of the subtype SVM classifier. The process of selecting subtype-specific genes followed the differential analysis procedure described in the Methods, with genes retained based on criteria of p.adjust < 0.001 and |log2FC| > 1. The mean interval was defined as [*μ*−*σ*, *μ*+*σ*], where *μ* represents the average expression of the gene in the patient cohort, and *σ* represents the standard deviation of gene expression in the patient cohort. (B) Predictive results of the SVM_10 model on the validation set. (C) ROC curve of the SVM_10 model on the training set. (D) ROC curve of the SVM_10 model on the validation set. (E) Expression distribution of the 10 classification genes across these two subtypes. (F) Heatmap of the expression levels of the 10 classification genes in different subtypes and normal samples.

In China, liver lesion biopsy is essential for HCC treatment and prognosis determination. To translate research findings into clinical applications, we used machine learning to identify diagnostic markers for HCC subtypes and built corresponding diagnostic models (Fig 5A). Specifically, we first selected differentially expressed genes in the subtypes (p.adjust < 0.001,|log2FC| > 1). Next, we filtered out genes with overlapping expression patterns between the subtypes and utilized elastic net regularization to identify informative genes for classification. From the elastic net results, we identified 10 significant genes for classification. Using a support vector machine (SVM), we created the SVM_10 classification model with 70% of the data for training and 30% for validation. SVM_10 achieved 87% accuracy on the validation set, with high AUC values confirming its reliability and effectiveness (Fig 5B–5D and Table 2).

**Table 2:** Classification results of subtype classifiers in the validation set.

Model	ACC	SEN	SPE	MCC
SVM_10	87%	94.79 2%	82.5%	0.7806
SVM_TTK	84%	87.3%	80.8%	0.644

Several studies provided evidence linking the dysregulated expression of these 10 classifier genes to adverse outcomes in HCC, including poor prognosis, increased proliferation, metastasis, and recurrence [43–50]. Notably, these genes showed significant expression differences between different subtypes and normal samples (Fig 5E and 5F), indicating their crucial involvement in HCC progression and potential as therapeutic targets. Among these genes, *TTK* has related drug information in the DrugBank database (https://go.drugbank.com/), indicating its potential as a therapeutic marker for HCC subtypes.

To assess the independent classification performance of *TTK*, we developed an additional SVM classifier, SVM_TTK, using *TTK* expression values. SVM_TTK demonstrated good classification performance, with 84% accuracy on the validation set (S7A Fig and Table 2). It also showed high AUC values on the validation and training sets (0.91 and 0.88, respectively, S7B and S7C Fig). SVM_TTK effectively classified patients from the Fudan cohort (n = 225, GEO accession number: GSE14520) [42] into two subtypes with significant survival differences (S8A Fig). These subtypes showed distinct *TTK* gene expression patterns (Mann-Whitney U test, S8B Fig), highlighting *TTK* as a potential diagnostic marker for HCC.

### Clinical and prognostic characteristics of HCC subtypes with interactive survival prediction Tool

The two subtypes showed significant differences in clinical characteristics such as gender, age, and tumor stage (chi-square test, S4 Table). Patients with CLASS A had a better prognosis and mainly exhibited lower alpha-fetoprotein (AFP) expression levels in early-stage samples (Mann-Whitney U test). On the other hand, CLASS B had a higher proportion of mid to late-stage samples, higher AFP expression levels, and a higher frequency of viral infection.

**Fig 6:**
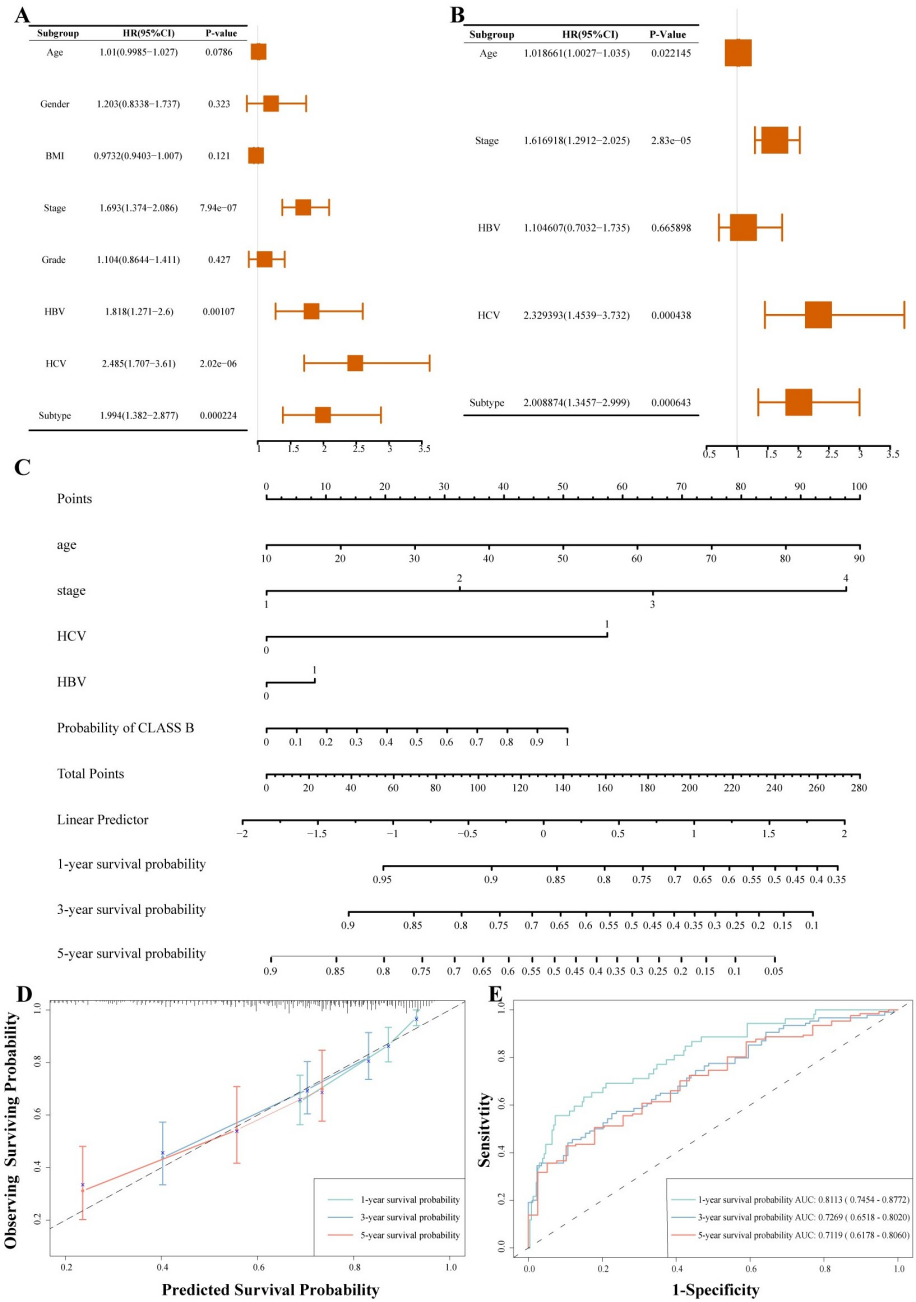
Construction and Evaluation of Clinical Prognostic Model. (A) Univariate Cox analysis of clinical features and subtypes in TCGA cohort. (B) Multivariate Cox analysis of clinical features and subtypes in TCGA cohort. (C) Nomogram model predicting HCC patients’ prognosis. (D) Calibration plot showing 1-, 3-, and 5-year survival probabilities for the nomogram model. (E) ROC curve evaluating predictive performance of the nomogram model in TCGA cohort.

Univariate and multivariate Cox regression analyses confirmed that the subtypes were independent prognostic factors for HCC patients (Fig 6A and 6B). Considering the lack of relatively comprehensive clinical information in the other two cohorts, we developed a multivariable prognostic model using the TCGA cohort. This model integrates age, viral infection, tumor staging, and the probability of CLASS B output by a machine learning diagnostic tool (Fig 6C). The C-index for this model is 0.698 (95% CI = 0.671 ~ 0.723), and the IBS is 0.166. Illustrated through a calibration plot, we demonstrate the model’s outstanding accuracy and predictive performance concerning 1, 3, and 5-year survival rates (Fig 6D and 6E).

Furthermore, decision curves indicated the superiority of the nomogram model over independent prognostic models using other predictors (S9 Fig). Additionally, we validated the model using the GSE14520 cohort [42] and achieved comparable AUC values (S8C Fig). To facilitate clinical application, we developed a user-friendly website allowing input of relevant information to automatically generate survival plots and probabilities (https://mike-wang-bjut.shinyapps.io/DynNomapp_HCC_Sutypes/).

### Analyzing subtype differences at the single-cell level

**Fig 7:**
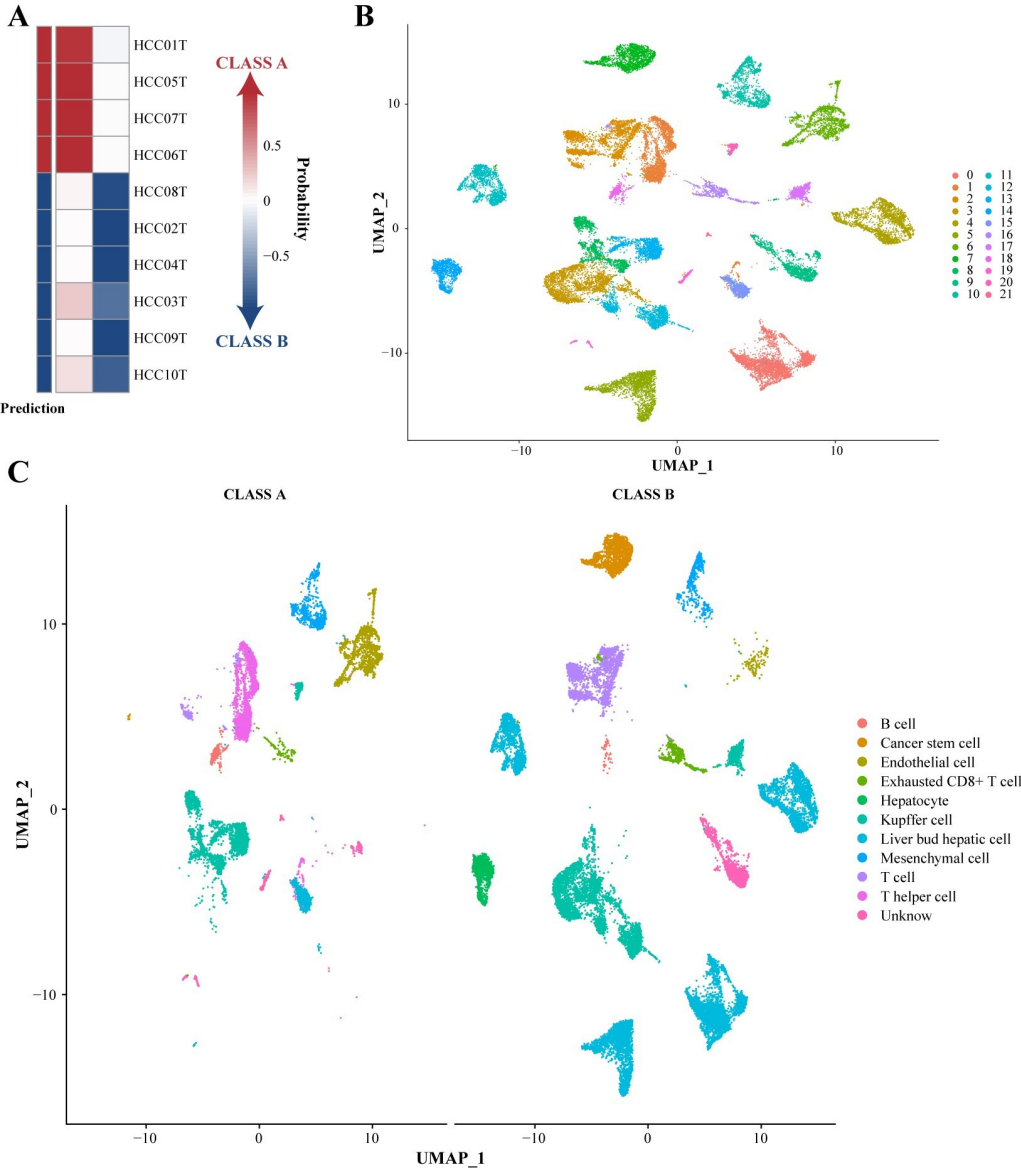
Single-cell analysis of HCC subtypes. (A) SVM_10 assigned subtypes to 10 primary HCC samples from GSE149614. (B) UMAP plot showing 21 cell clusters. (C) Cell type annotation in different subtypes using marker genes.

To understand the differences between these two subtypes at a higher resolution, we downloaded scRNA-seq data from 10 primary HCC patients from GSE149614 [51] and performed cellular-level QC. Pseudo-bulk data was created by summing gene expression across sample cells. After standardization, the data was classified into two subtypes using the SVM_10 model (Fig 7A).

Using the UMAP method, we identified 21 cell clusters, and each cluster was annotated through the gene enrichment analysis method (Fig 7B). The single-cell-level results strongly agreed with our bulk-level findings, indicating that CLASS B had enriched immune cells and cancer stem cells compared to CLASS A (Fig 7C). Moreover, T cells from CLASS B exhibited higher expression levels of four immune checkpoint genes, consistent with their differential expression at the bulk level (S10 Fig). These findings further prove that the CLASS B subtype may have a higher stemness phenotype and could be more responsive to targeted immune checkpoint therapies in HCC.

### Drug sensitivity differences across subtypes

**Fig 8:**
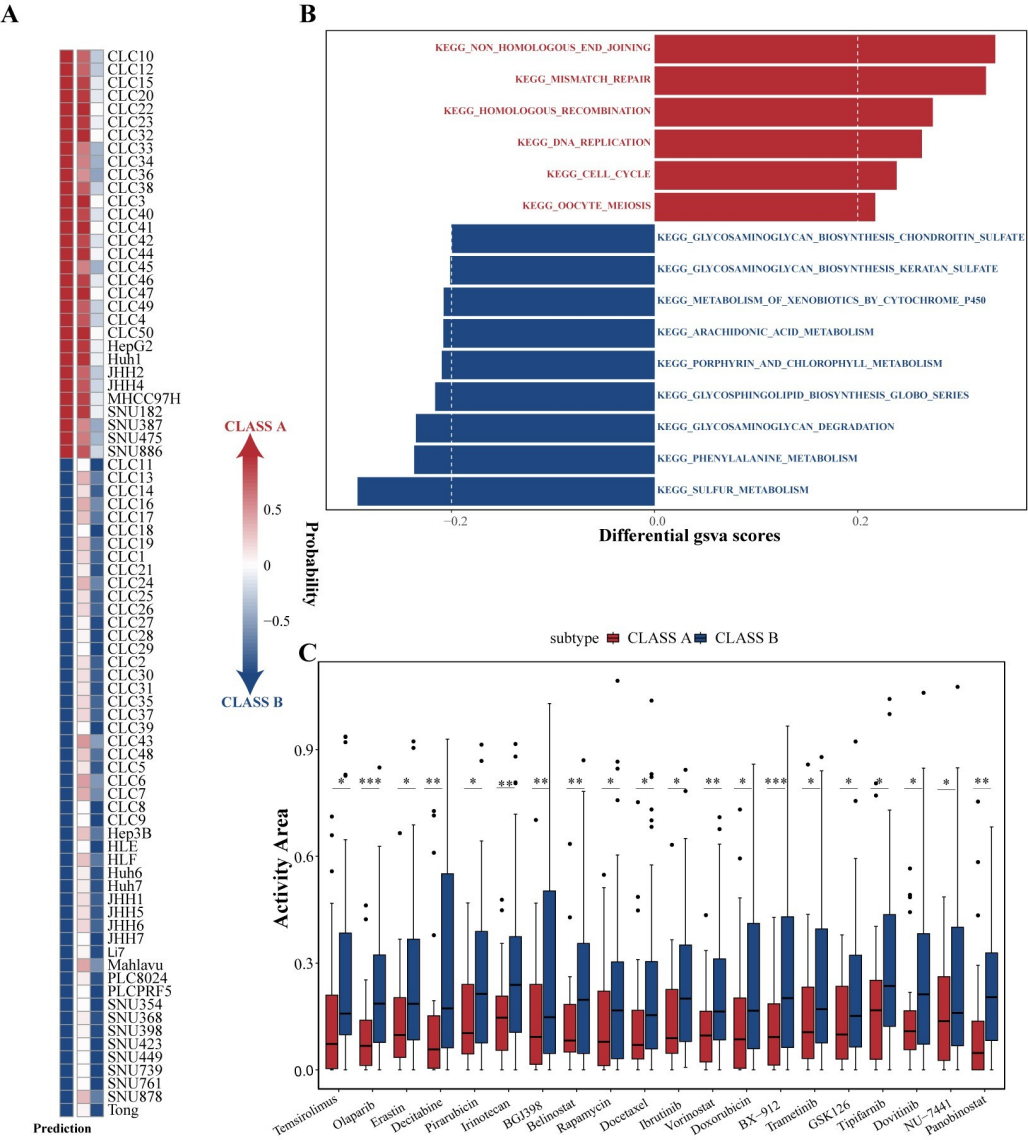
The drug sensitivity analysis of HCC subtypes. (A) The SVM_10 model assigned subtypes to 81 HCC cell lines from the LIMORE dataset. (B) KEGG enrichment analysis of the two subtypes. (C) Box plots illustrating drugs with differential activity area in the two subtypes.

Finally, to investigate the differences in drug sensitivity across subtypes, we applied the SVM_10 model to the LIMORE dataset, containing the mutation, RNA, and drug response data for 81 HCC cell lines [52]. The SVM_10 model successfully classified the cell lines into two subtypes, similar to the clinical patient subtypes (Fig 8A and 8B). CLASS B showed stronger sensitivity to cell proliferation inhibitors, such as Temsirolimus, Camptothecin, and BX-912, possibly related to its strong proliferative characteristics. (Fig 8C).

## Discussion

This study expanded HCC driver genes using gene families and identified protein domains with significant mutation burden, through permutation testing [12]. From the results, some of these domains were associated with classical cancer mutation events, such as RTK and PI3K/AKT signaling pathways, along with the SET domain, known for its methyltransferase activity crucial for maintaining the tumor-suppressive function of genes [53]. Moreover, the zf-H2C2_2 domain in zinc finger transcription factors also showed high mutation frequency and entropy, potentially leading to widespread transcriptional dysregulation in tumors and conferring a selective growth advantage to the tumor [10].

To overcome the discreteness inherent to mutation data for cancer stratification, we employed the NBS algorithm [27] to transform it into continuous features. These features were subsequently integrated with gene expression data using the SNF algorithm [28] for clustering analysis. The results demonstrated that SNF effectively captured the smoothed mutation features from NBS and utilized them for clustering. Through consensus clustering, we classified HCC into two subtypes, CLASS A and CLASS B, with significant differences in survival, with CLASS B displaying a lower survival probability. Moreover, compared with previous studies, our subtype model exhibits a higher value in clinical prediction.

Interestingly, only driver genes showed poor stability in clustering and survival differences compared to the stratification results obtained through gene family expansion. This may be attributed to insufficiently utilizing the entire stratification algorithm’s extensive protein-protein interaction network information when focusing solely on driver genes. In the stratification algorithm, gene family expansion allowed for a more comprehensive consideration of family members associated with driver genes. This approach introduced more relevant information, aiding in the revelation of complex molecular relationships and regulatory mechanisms. By incorporating family members into consideration, our understanding of the overall biological network was enhanced, resulting in more biologically reasonable stratification outcomes.

Subsequently, we conducted further analysis of the two subtypes. GSVA results indicated that CLASS A exhibited prominent metabolic features enriched in pathways such as organic acid metabolism, redox reactions, fatty acid metabolism, and glycolysis. Conversely, CLASS B was enriched in proliferative pathways, including mitosis and the cell cycle. Interestingly, we did not explicitly emphasize metabolism-related genes in the classifier genes. Therefore, the heightened metabolic features in CLASS A suggest that aberrant changes in specific metabolic genes may contribute to the progression of HCC.

Moreover, the increased dependency on these metabolic pathways may lead to metabolic vulnerability, implying the potential therapeutic efficacy of inhibiting these pathways in treating CLASS A tumors [54]. Meanwhile, CLASS B exhibited higher immune cell abundance and overexpression of immune checkpoint molecules such as CTLA4 and HCVAR2, indicating its potential suitability for immune checkpoint inhibitor therapy. Additionally, CLASS B had higher stemness scores, implying a more active population of cancer stem cells and increased tumor cell dedifferentiation [38]. These findings were confirmed in subsequent single-cell analysis.

Next, we compared genomic differences between the subtypes and observed significant chromosomal instability in CLASS B, characterized by deletions in chromosomes 4p, 4q, 13q, 16p, 16q, and 17p. These deletions are frequently found in clinically advanced, poorly differentiated, large, and metastatic HCC cases [55,56]. Additionally, our subtypes demonstrated a high degree of consistency with other subtypes. For example, CLASS A was enriched in several high metabolic subtypes with a better prognosis, such as Hosdia S3, iHCC1, and hALDH2. Additionally, CLASS B was enriched in low metabolic flux, high *TP53* mutation, and highly proliferative subtypes, such as Hosdia S1, Hosdia S2, and iHCC3. In addition, referring to the findings of Benfeitas et al. [30], these two subtypes may employ different mechanisms to resist reactive oxygen species (ROS). Notably, during the subtype stratification of HCC, we incorporated mutation data. We observed significant differences in the mutation frequencies of classical oncogenes, such as *RB1*, *ALB*, *APOB*, and *NFE2L2*, among others, between the two distinct subtypes, except for *TP53* and *CTNNB1*. This difference was not significant among other subtypes (p > 0.05).

*RB1*, a crucial tumor suppressor gene, regulates the cell cycle by inhibiting E2F transcription factors and cyclin-dependent kinases during G1 to S phase transition [57,58]. *RB1* mutations were predominantly found in CLASS B samples (Fisher’s exact test, p = 1.39×10–8), explaining the heightened proliferative features in CLASS B. The Wnt-β-catenin signaling pathway is frequently activated in HCC [59]. Aggressive HCC subtypes, unlike well-prognosed ones, enhance the Wnt pathway by regulating intracellular free β-catenin through TGF-β overexpression [31]. In CLASS B, we observed the RGS domain primarily occurring in *AXIN1* (Fisher’s exact test, p = 0.012632, S11 Fig and S7 Table). This domain binds to APC protein, contributing to β-catenin degradation [60]. Missense mutations in the RGS domain of *AXIN1* promote *AXIN1* aggregation, leading to impaired β-catenin degradation [61]. Thus, in this aggressive subtype, gene mutations may indirectly or directly affect β-catenin degradation, resulting in Wnt pathway activation.

Finally, we developed a 10-gene SVM subtypes classifier between the subtypes. An intriguing finding was made regarding *TTK*, one of the classifier genes with relevant drug records in Drugbank. *TTK* itself demonstrated excellent performance in subtype classification. *TTK*, also known as Mps1, recruits other SAC proteins to unattached kinetochores during prophase to activate SAC-related arrest [62]. Inhibiting *TTK* activity leads to premature chromosome segregation, severe chromosomal missegregation, aneuploidy, and cell death [63]. *TTK* is overexpressed in various human tumors, including HCC. Studies indicate its role in promoting HCC cell malignancy [64]. *TTK* inhibitors like BOS172722 [65] and CFI-402257 [66] have shown promise in cancer treatment, making *TTK* a potential independent diagnostic biomarker and therapeutic target for specific HCC subtypes.

Although we have identified two HCC subtypes with distinct survival and biological characteristics using driver genes and their family members, there are some limitations. Firstly, the lack of patient cohorts with comprehensive multi-omics and clinical data restricts the validation of the subtype models across diverse populations. Secondly, this study needs to include relevant in vivo/in vitro experiments to demonstrate the therapeutic effect of relevant inhibitors on specific subtypes.

## Conclusion

In conclusion, we successfully constructed two HCC subtypes with significant differences in survival. We have delved into the biological disparities underlying these subtypes by integrating diverse omics data. Our tailored prognostic and classification models demonstrated robustness and accurately predicted overall survival in HCC patients. Notably, *TTK* emerged as a crucial diagnostic biomarker and potential therapeutic target for specific HCC subtypes. Our study offers a fresh outlook on HCC subtypes, enhancing our understanding of the disease’s pathogenesis and potential therapeutic strategies.

## Methods

### Data sources and preprocessing

This study included a cohort of 893 patients with primary tumors from three HCC datasets, namely TCGA_LIHC, ICGC_JP, and ICGC_FR. Among these, 690 patients (S8 Table) were retained for subsequent analysis as they had both RNA-seq and mutation data available.

Somatic mutations, miRNA expression, HM450 methylation, copy number variants, and clinical information for the TCGA_LIHC cohort were obtained from the GDC Data Portal(https://portal.gdc.cancer.gov/), mRNA-Seq data were obtained from the TCGAxGETx combined dataset from UCSC Xena (http://xena.ucsc.edu/). Somatic mutations, mRNA-Seq, and clinical data for ICGC_JP and ICGC_FR cohorts were obtained from the ICGC database (https://dcc.icgc.org/).

We standardized them into TPM values to ensure consistency and comparability of RNA-seq data across the three cohorts. We mitigated batch effects using the "ComBat" function within the R package "sva" [67]. After amalgamating these cohorts into a single merged cohort, we filtered out genes exhibiting TPM values ≤ 0 in over 30% of the samples. This preliminary data processing step effectively reduced noise, ensuring data quality by retaining approximately 17,660 genes for subsequent analyses.

Methylation probes were preprocessed and normalized using the R package "ChAMP" [68]. We removed null rate probes in more than 30% of methylation and miRNA data samples, using KNN (K = 15) interpolation with the R package "impute".

For somatic mutation data, we retained only non-silent mutations in the coding region.

### Protein domain with significant mutation burden

Before the domain mutation analysis, we further processed the somatic mutation data. Samples with tumor mutational burden (TMB) values >30 mutations/Mb were excluded to eliminate the influence of hypermutated samples. We calculated TMB as the count of non-silent mutations within coding region exons divided by 38 Mb. We also removed MAF entries with non-point mutations, amino acid change positions larger than the protein length, and the reference amino acids not aligning with the protein reference sequence.

A permutation test was used to identify protein domains with significant mutation burden, as described by Miller et al. [12] In this test, we assumed that all amino acids within the protein have an equal chance of mutation. The permutation test assessed whether the observed values significantly deviated from the empirical distribution obtained from random mutations in the gene. The P-value after i permutations was defined as:

$$p.value=\frac{Number\;larger\;than\;the\;observed\;value+1}{i+1}$$

To quantify the information about the distribution of the mutation burden within a specific domain among its gene members, we computed the entropy value (*S*). The entropy is defined as:

$$S=\frac{-\sum_{i=1}^{n}P\left(x_{i}\right)lnP\left(x_{i}\right)}{\text{ln}\;n}$$

In information entropy theory, the entropy value reaches its maximum when the distribution is uniform. Thus, the entropy value is close to 1 if mutations in the domain are uniformly distributed across the family genes. Conversely, if the mutations are not uniformly distributed, the entropy value will be closer to 0.

### Differential analysis

We used the Mann-Whitney U test for miRNA and mRNA data to find genes with expression differences between different groups. We corrected the p-values using the Benjamin-Hochberg method. We define differentially expressed miRNA (DMi) and differential gene (DEG) for those with | Log2FC |>1 and p.adjust<0.05. Additionally, we excluded DMi and DEG with mean expression levels less than 1 in both the test and control groups.

Differential methylation probes (DMPs) were identified using the R package "ChAMP" for DNA methylation data, considering probes with |Δβ| > 0.2 and p.adjust < 0.05 as DMPs.

For copy number data, we employed the GISTIC2.0 function available in GenePattern(https://www.genepattern.org/) to identify chromosomal regions and genes exhibiting significant copy number variations.

### Define driver-dysregulated gene

We defined driver-dysregulated genes (DDGs) as genes that exhibit transcriptional dysregulation due to alterations in DNA methylation, miRNA expression, or copy number variation. To comprehensively define DDGs, we further divide DMPs into three groups: distal enhancers, promoters (TSS1500, TSS200, 5’UTR, and 1stExon), and gene body. Here, TSS1500 refers to the region 200–1500 bases upstream of the transcriptional start site (TSS), while TSS200 represents the region 0–200 bases upstream of the TSS. The R packages "ELMERv.2" [69] and "ChAMP" were used to identify distal enhancer probes, promoter and gene body methylation probes, and their target genes. For DMi, we used the R package "multiMiR" [70] to obtain target genes based on experimental or computational predictions. Genes with a copy number gain or loss ratio greater than 20% were categorized as gain or loss groups, respectively (CNV levels greater than 1 for gain and less than -1 for loss). The association between DMPs, DMi, CNV, and DEG was analyzed using Spearman correlation (threshold: |ρ| > 0.2, p-value after Benjamin Hochberg correction < 0.05). Venn diagrams were used for result filtering to illustrate the relationships between different omics layers and the transcriptome.

### Subtype recognition model

In this paper, we aimed to construct HCC subtypes using somatic mutation data and mRNA expression data. First, we converted the mutation MAF file into a binary matrix, where 0 represents no mutation, and 1 indicates a mutation presence in that sample. To smooth the mutation data, we used pyNBS (https://github.com/idekerlab/pyNBS) [71], a Python version of the Network-based stratification (NBS) algorithm [27]. This algorithm employs network propagation to smooth the mutation signals, enhancing their classification capabilities like other continuous features. The following formula can represent the process of network propagation:

$$F_{t+1}=\alpha F_{t}A+\left(1-\alpha \right)F_{0}$$

*F*_*0*_ represents the patient mutation matrix, *A* represents the normalized adjacency matrix of the gene interaction network, and *α* is an adjustment parameter that controls the distance allowed for mutation signals to spread through the network during propagation. The propagation function iterates until convergence (determined by the matrix norm of *F*_*t+1*_- *F*_*t*_ < 1×10^−6^), with *α* set to 0.7 following recommendations from the references for smoothing the mutation data.

The gene expression data underwent log_2_(x+1) transformation and Z-score normalization. Next, we integrated the smoothed mutation data with the gene expression data to form a similarity matrix *W* using the R package "SNFtool". For integration, we used 20 nearest neighbors, set the variance of the local model to 0.5, and performed 20 iterations of the diffusion process.

We conducted consensus clustering using the similarity matrix W with the R package "ConsensusClusterPlus." We set clustering parameters as follows: maximum clusters = 6, iterations = 5000, item sample proportion = 0.8, distance metric = ’spearman’, and clustering algorithm = ’hc’. We evaluated clustering quality using the silhouette coefficient (*Sil*), defined as follows

$$Sil\left(i\right)=\frac{b\left(i\right)-a\left(i\right)}{\text{max}\left\{a\left(i\right),b\left(i\right)\right\}}$$

Here, *a(i)* represents the average distance between vector i and all other points within the same cluster. In contrast, *b(i)* represents the minimum average distance between vector i and all points in a cluster that does not include it.

### Gene enrichment analysis

The R package "clusterProfiler" [72] performs hypergeometric distribution tests on the annotations of specific gene sets in different databases.

To convert gene expression data into scores for particular biological processes, we utilized the R packages "GSVA" [73] and "msigdbr." Subsequently, a Student’s t-test was employed to identify significant differences in biological processes among different subtypes. A differential biological process was defined as |ΔGSVA score| > 0.2 and Benjamini-Hochberg adjusted p < 0.05.

### CIN ratio

Based on previous research, we assessed chromosomal instability (CIN) across different subtypes using the CIN ratio [74,75]. To calculate the CIN ratio for each tumor sample, we extracted the "broad_values_by_arm.txt" file from the GISTIC2.0 results. In this file, CNV scores exceeding 0.1 or falling below -0.1 were regarded as alterations. The CIN ratio was defined as:

$$CIN\;ratio=\;\frac{Number\;of\;chromosomal\;arm\;abnormalities}{Total\;number\;of\;chromosome\;arms}$$

### Nomogram model of HCC subtypes

We used the R package "survival" to construct univariate and multivariate Cox prognostic models. The "forestplot" package was employed to generate forest plots, while the "rms" package facilitated the creation of a nomogram for the multivariate model. To assess the performance of the nomogram model, we employed calibration plots, decision curve analysis, and receiver operating characteristic (ROC) curves, leveraging the R packages "rms," "dcurves," and "timeROC" respectively. Additionally, the R package "DynNom" wasutilized to develop a dynamic nomogram model and an interactive webpage.

### Single-cell data processing

We downloaded the HCC single-cell RNA sequencing (scRNA-seq) data from the GEO database (GEO accession: GSE149614 [51]). For further analysis, we selected 10 primary tumor samples. Quality control (QC) was performed using the R package "Seurat" [76], involving cell-level QC and gene-level QC. Specifically, cells with UMIs greater than 500, expressing genes between 500 and 8000, and mitochondrial content less than 10% were retained. We kept genes with expression data in at least 10 cells for gene-level QC. Subsequently, 22,298 genes and 31,490 cells were used for subsequent analyses.

### Single-cell data dimensionality reduction clustering

We processed the scRNA-seq data using the R package "Seurat." Firstly, the "NormalizeData" function was applied for background correction and normalization. We employed the "FindVariableFeatures" function (selection method = ’vst’, ’ x-axis cut off = (0.0125, 3), y-axis cut off > 0.5) to identify the top 2000 highly variable genes. Scaling of the data was carried out with the "ScaleData" function, excluding mitochondrial contamination heterogeneity. Next, we reduced data dimensionality with the "RunPCA" function, selecting the top 30 principal components based on the "ElbowPlot" analysis. Cell clustering was conducted with a resolution of 0.2 using the "FindClusters" function, and cell clusters were visualized with the "RunUMAP" and "DimPlot" functions. Significant marker genes in different clusters were identified using the "FindAllMarkers" function. To annotate the cell clusters, we adopted a statistical-based approach by performing enrichment analysis on the marker genes using the "clusterProfiler" R package. We downloaded the Human cell markers dataset from CellMarker (http://biocc.hrbmu.edu.cn/CellMarker).

### Subtype machine learning model

All machine learning classifiers were constructed using the "scikit-learn" package (version 1.0.2) in Python (version 3.9.12). Cross-validation and grid search were used to obtain the best hyperparameters for the model. To assess the performance of the classification results, we employed Confusion matrices, ROC curves, accuracy (ACC), sensitivity (SEN), specificity (SPE), and Matthews correlation coefficient (MCC). These evaluation metrics are defined as follows:

$$ACC=\frac{TP+TN}{TP+TN+FP+FN}\times 100\text{\%}$$

$$SEN=\frac{TP}{TP+FN}\times 100\text{\%}$$

$$SPE=\frac{TN}{TN+FP}\times 100\text{\%}$$

$$MCC=\frac{\left(TP\times TN\right)-\left(FP\times FN\right)}{\sqrt{\left(TP+FN\right)\times \left(TN+FP\right)}\times \left(TP+FP\right)\times \left(TN+FN\right)}\times 100\text{\%}$$

Here, *TP* denotes the count of true positives, *TN* denotes the count of true negatives, *FP* denotes the count of false positives, and *FN* denotes the count of false negatives. The machine learning model can be found at https://github.com/Mike-W29/SVM_model_for_HCC_subtype.

### Statistic analysis

Statistical analyses for this paper were conducted using R (version: 4.2.1). Kaplan-Meier curves were evaluated with the log-rank test to determine significance. The chi-square and Fisher’s exact tests explored associations between clinical characteristics in different groups. Unless otherwise specified, statistical significance was defined as p-values < 0.05 (two-tailed). In the paper, significance levels were denoted as * (p < 0.05), ** (p < 0.01), *** (p < 0.001), **** (p < 0.0001), and "na" indicated no statistical difference.

## Supporting information

S1 Fig Identification of driver gene-related subtypes in the TCGA cohort. (A) Silhouette coefficients for different values of k. (B) Silhouette plot for k = 2. (C) Consensus matrix heatmap defining two subtypes. (D) Five-year survival curves for the two subtypes, with CLASS A represented in red and CLASS B in blue.
(TIF)

S2 Fig The Sankey diagrams depicting the associations between the subtypes identified in our study and those from previous research studies. (A) Hoshida 3-class subtypes. (B) Bidkhori 3-class subtypes. (C) Benfeitas 2-class subtypes. (D) TCGA 3-class subtypes.
(TIF)

S3 Fig C-index analysis comparing the subtypes identified in this study with previous research. (TIF)

S4 Fig XCELL analysis results for the two subtypes. (A) Analysis of different cell abundances between the two subtypes using XCELL. The statistical significance of the differences was assessed using the Mann-Whitney U test. (B) Analysis of different immune scores, stromal scores, and microenvironment scores between the two subtypes using XCELL. The statistical significance of the differences was assessed using the Mann-Whitney U test.
(TIF)

S5 Fig Box plots depicting the expression of *KRT19* in the two subtypes. Statistical significance of the differences was assessed using the Mann-Whitney U test to determine if these differences were statistically significant.
(TIF)

S6 Fig Box plot showing the TMB in each subtype. (TIF)

S7 Fig The classification results of the HCC subtypes using the SVM_TTK model. (A) Predicted results of the SVM_TTK model on the validation set. (B) ROC curve of the SVM_TTK model in the training set. (C) ROC curve of the SVM_TTK model in the validation set.
(TIF)

S8 Fig The validation of the classification and prognostic models. (A) Survival analysis of the GSE14520 cohort based on the classification results using the SVM_TKK model. (B) Expression levels of *TTK* in the two subtypes of the GSE14520 cohort, with statistical significance determined by the Mann-Whitney U test (C) ROC curves depicting the predictive results of the prognostic model for 1-, 3-, and 5-year survival probabilities in the GSE14520 cohort.
(TIF)

S9 Fig The decision curve analysis for the prognostic model of the subtypes. (A) Decision curve analysis for 1-year survival. (B) Decision curve analysis for 3-year survival. (C) Decision curve analysis for 5-year survival.
(TIF)

S10 Fig The single-cell expression profiles of four immune checkpoint genes that exhibit differential expression at the bulk level. (TIF)

S11 Fig Oncoplot illustrating the specific differences in protein domain mutation frequencies between the two subtypes (chi-square test). (TIF)

S1 Table Driver gene and their family number. (XLS)

S2 Table Protein domains with significant mutations. (XLS)

S3 Table List of stratification genes. (XLS)

S4 Table Differential clinical features between the two subtypes. (XLS)

S5 Table HCC subtypes from other studies used in this paper. (XLS)

S6 Table Differential enrichment analysis of two subtypes in REACTOME gene sets using GSVA. (XLS)

S7 Table Protein domains with specific differences in mutation frequencies between the two subtypes. (XLS)

S8 Table Description of the patient cohort. (XLS)
