# Supplementary figures and images for: Machine learning and multi-omics data reveal driver gene-based molecular subtypes in hepatocellular carcinoma for precision treatment
Source: PLoS Comput Biol. 2024 May 10;20(5):e1012113. doi: 10.1371/journal.pcbi.1012113 (PMC11230636; doi:10.1371/journal.pcbi.1012113)

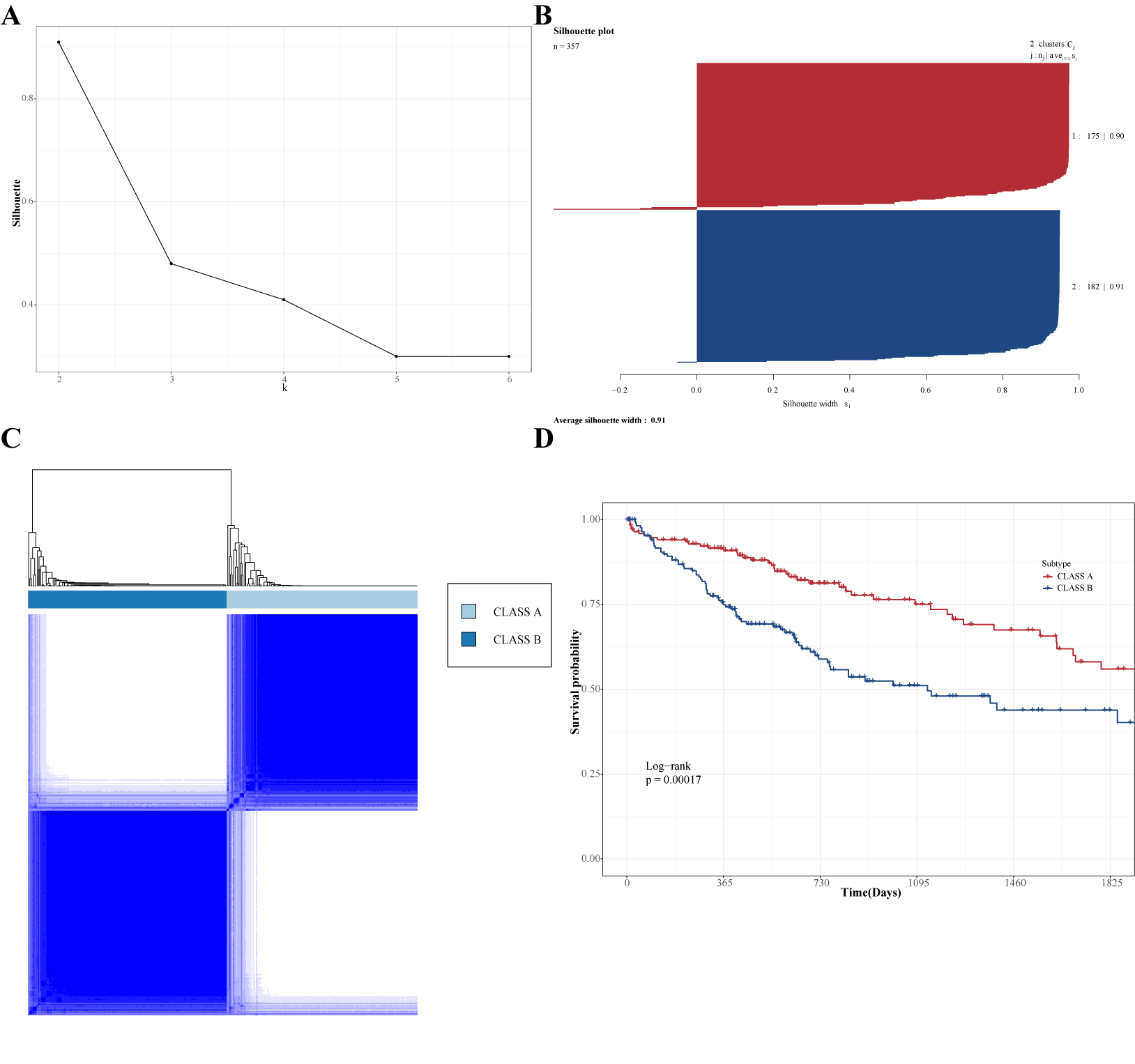

Supplement: S1 Fig — (A) Silhouette coefficients for different values of k. (B) Silhouette plot for k = 2. (C) Consensus matrix heatmap defining two subtypes. (D) Five-year survival curves for the two subtypes, with CLASS A represented in red and CLASS B in blue. (TIF) [file pcbi.1012113.s001.tif]

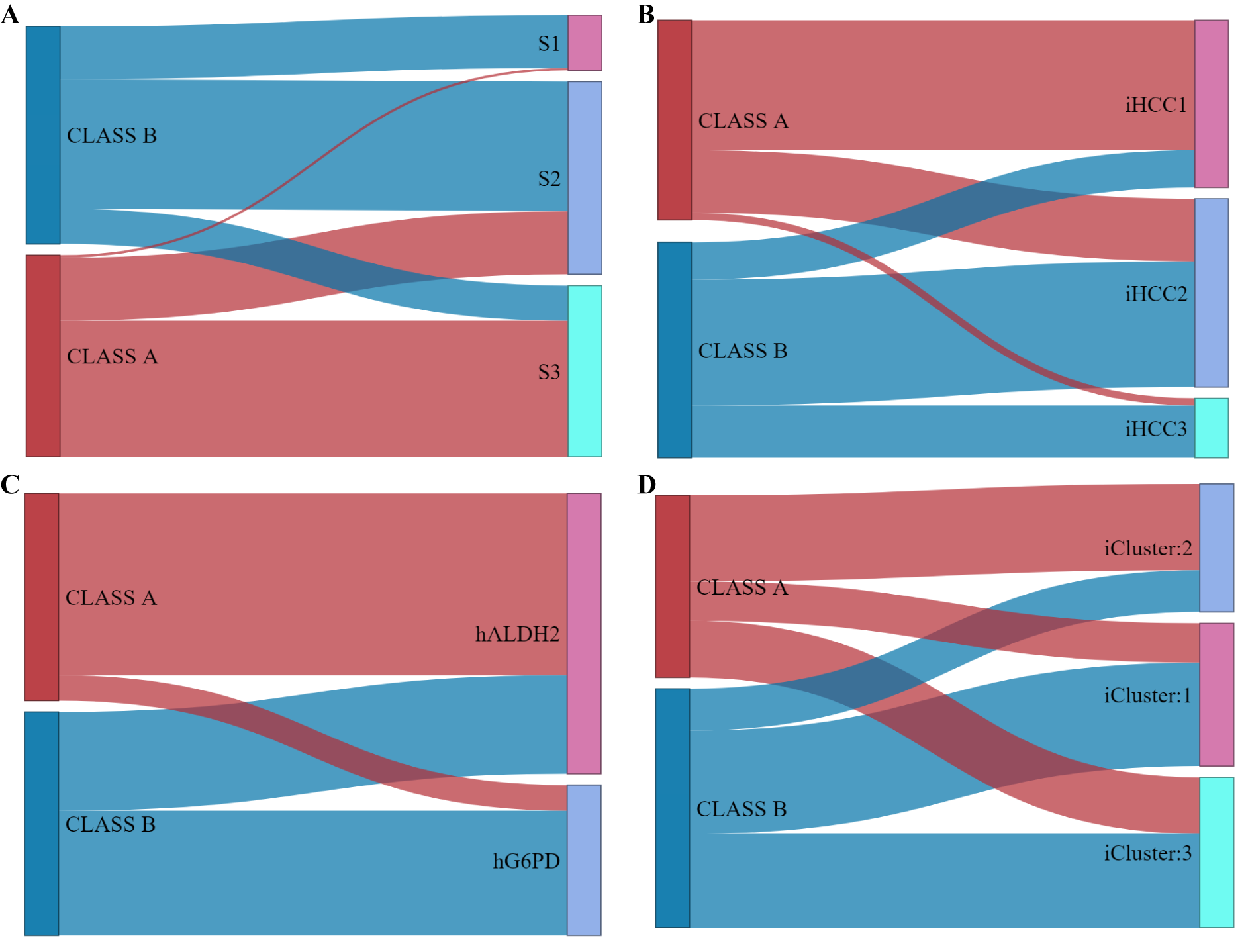

Supplement: S2 Fig — (A) Hoshida 3-class subtypes. (B) Bidkhori 3-class subtypes. (C) Benfeitas 2-class subtypes. (D) TCGA 3-class subtypes. (TIF) [file pcbi.1012113.s002.tif]

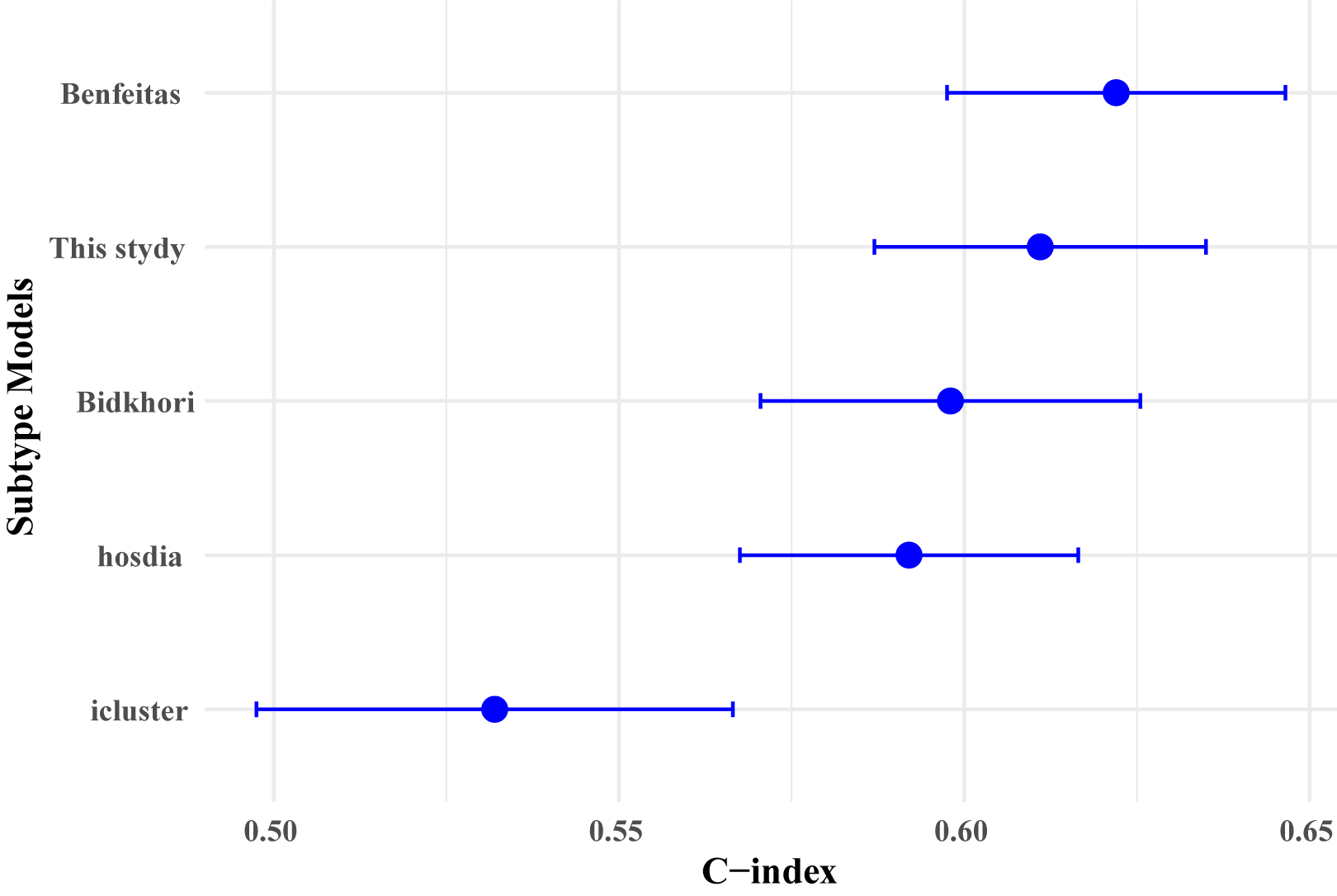

Supplement: S3 Fig — (TIF) [file pcbi.1012113.s003.tif]

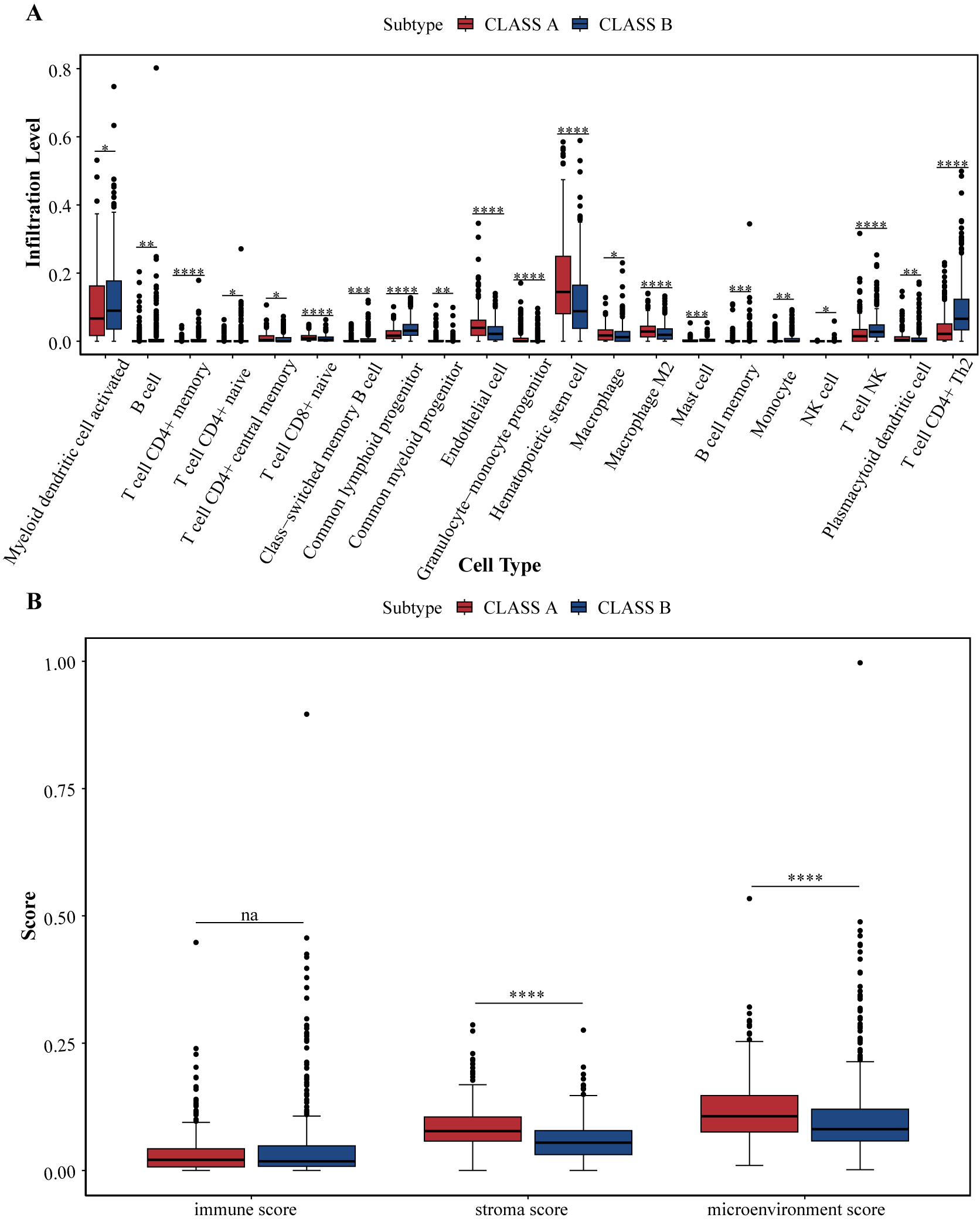

Supplement: S4 Fig — (A) Analysis of different cell abundances between the two subtypes using XCELL. The statistical significance of the differences was assessed using the Mann-Whitney U test. (B) Analysis of different immune scores, stromal scores, and microenvironment scores between the two subtypes using XCELL. The statistical significance of the differences was assessed using the Mann-Whitney U test. (TIF) [file pcbi.1012113.s004.tif]

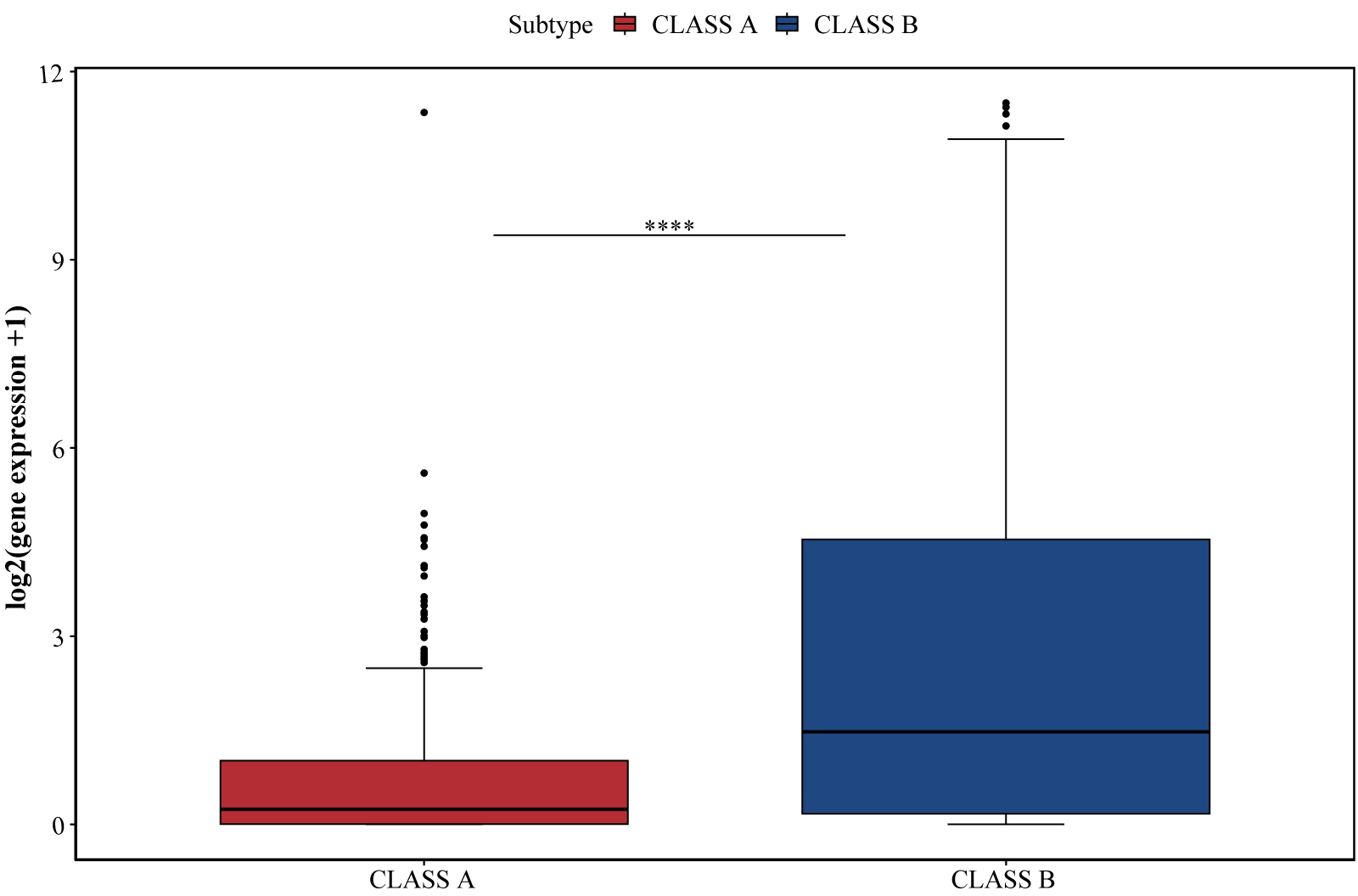

Supplement: S5 Fig — Statistical significance of the differences was assessed using the Mann-Whitney U test to determine if these differences were statistically significant. (TIF) [file pcbi.1012113.s005.tif]

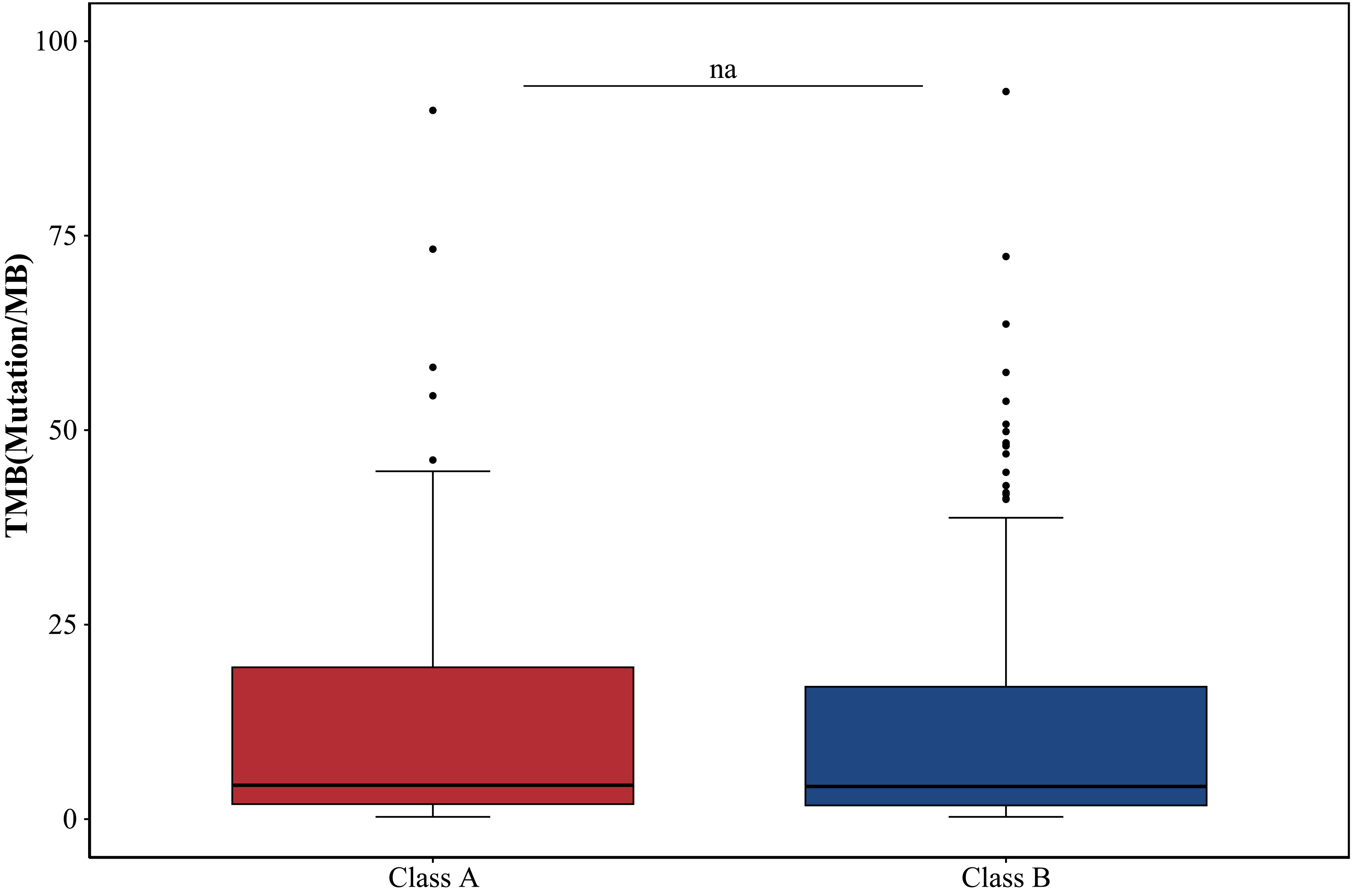

Supplement: S6 Fig — (TIF) [file pcbi.1012113.s006.tif]

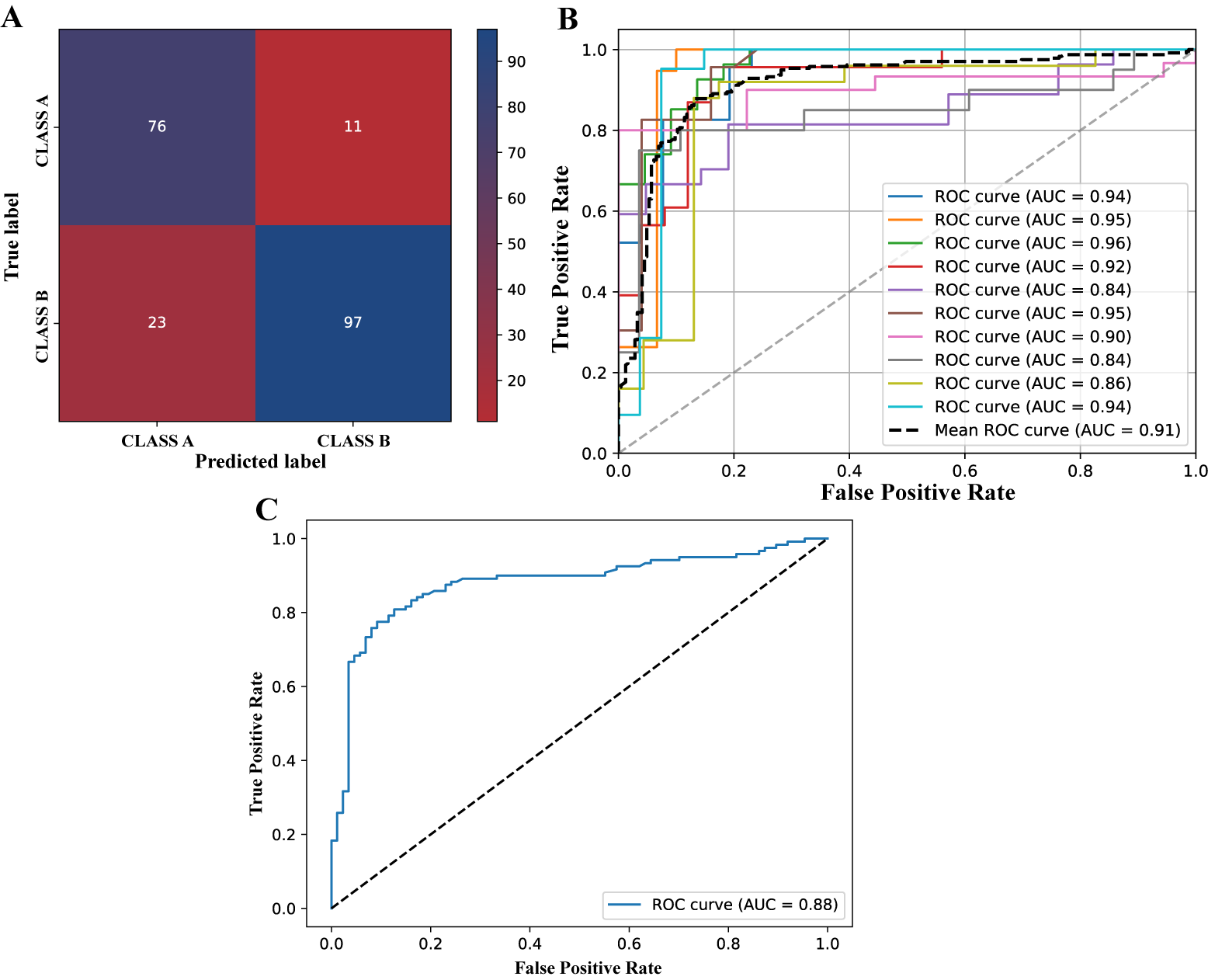

Supplement: S7 Fig — (A) Predicted results of the SVM_TTK model on the validation set. (B) ROC curve of the SVM_TTK model in the training set. (C) ROC curve of the SVM_TTK model in the validation set. (TIF) [file pcbi.1012113.s007.tif]

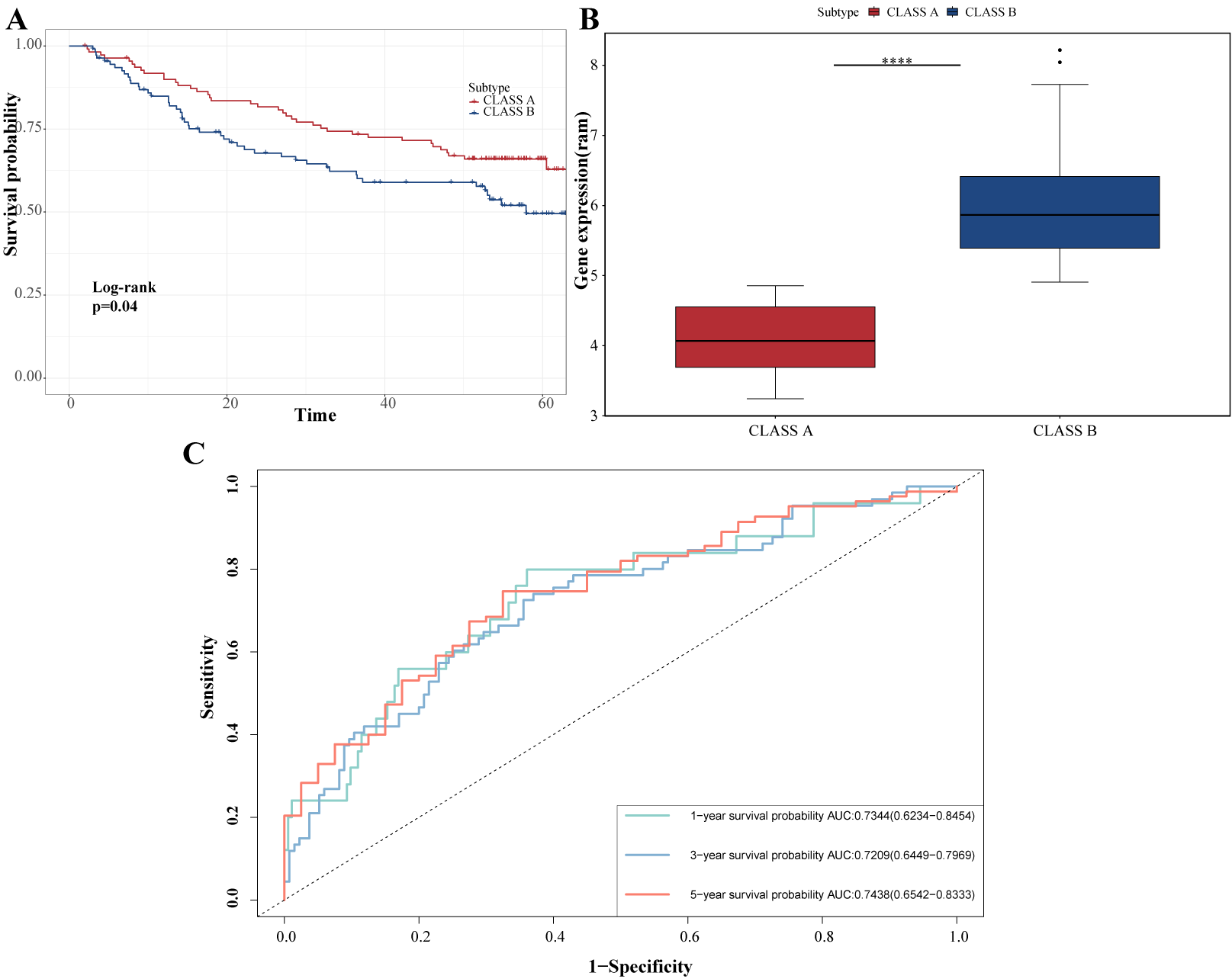

Supplement: S8 Fig — (A) Survival analysis of the GSE14520 cohort based on the classification results using the SVM_TKK model. (B) Expression levels of TTK in the two subtypes of the GSE14520 cohort, with statistical significance determined by the Mann-Whitney U test (C) ROC curves depicting the predictive results of the prognostic model for 1-, 3-, and 5-year survival probabilities in the GSE14520 cohort. (TIF) [file pcbi.1012113.s008.tif]

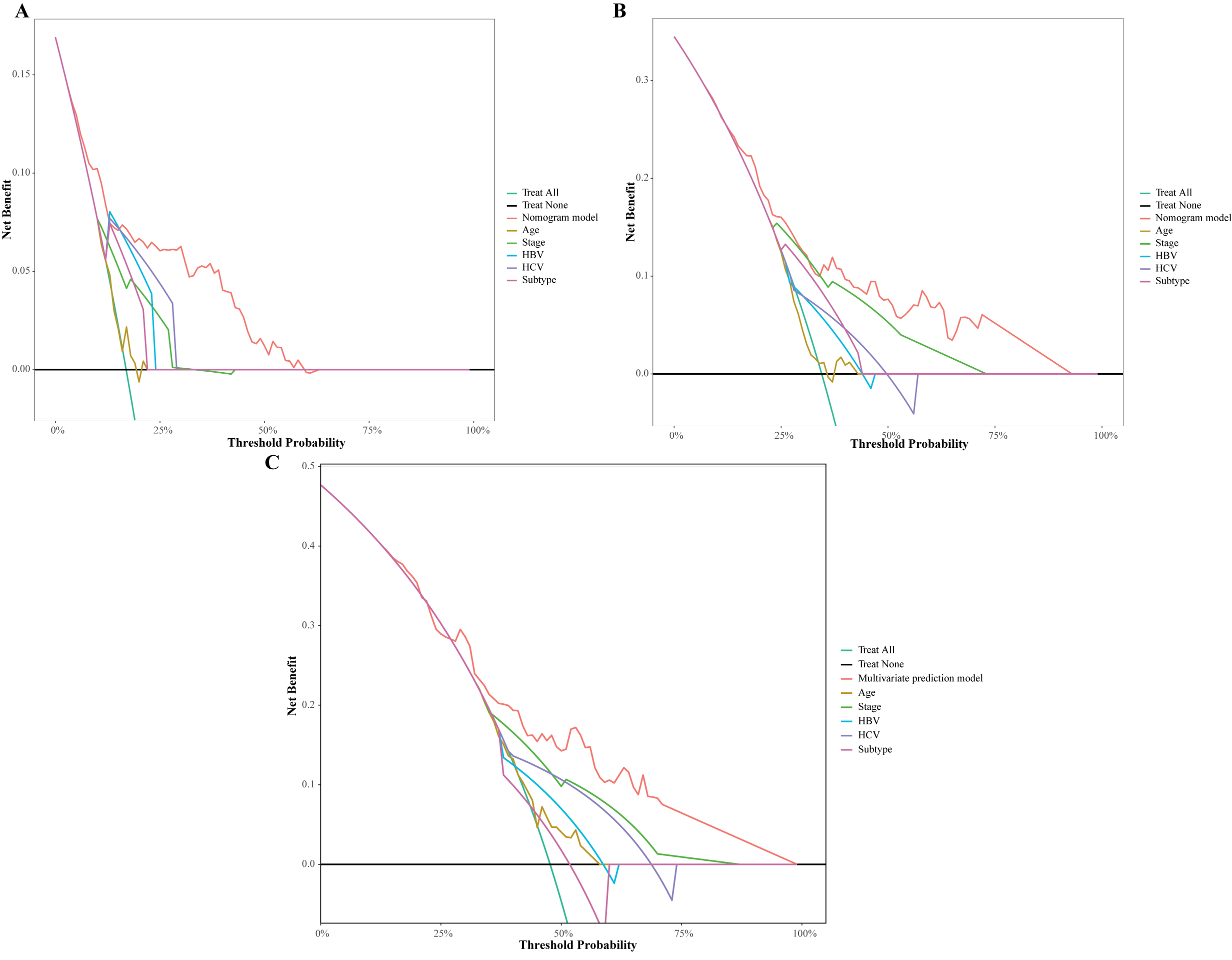

Supplement: S9 Fig — (A) Decision curve analysis for 1-year survival. (B) Decision curve analysis for 3-year survival. (C) Decision curve analysis for 5-year survival. (TIF) [file pcbi.1012113.s009.tif]

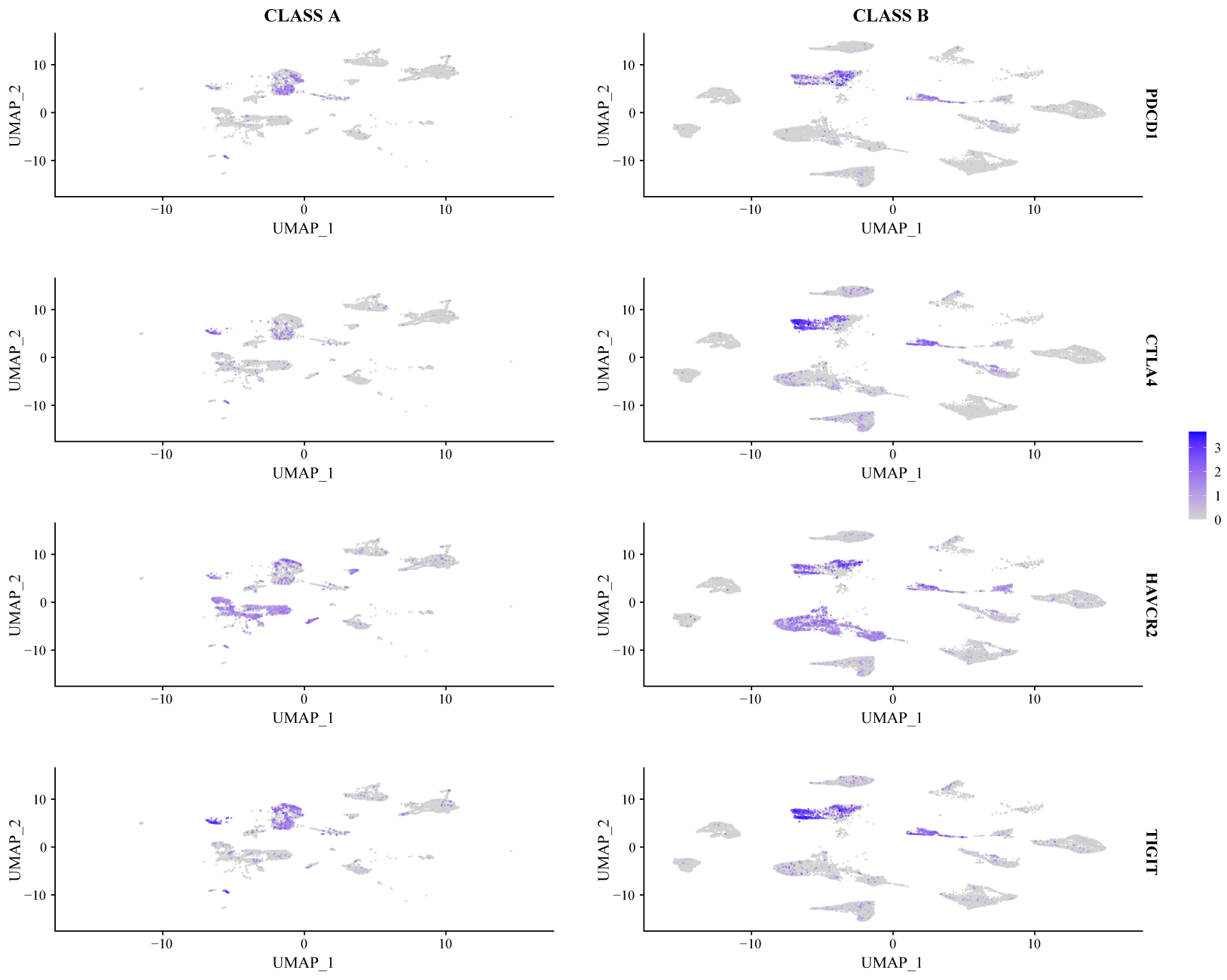

Supplement: S10 Fig — (TIF) [file pcbi.1012113.s010.tif]

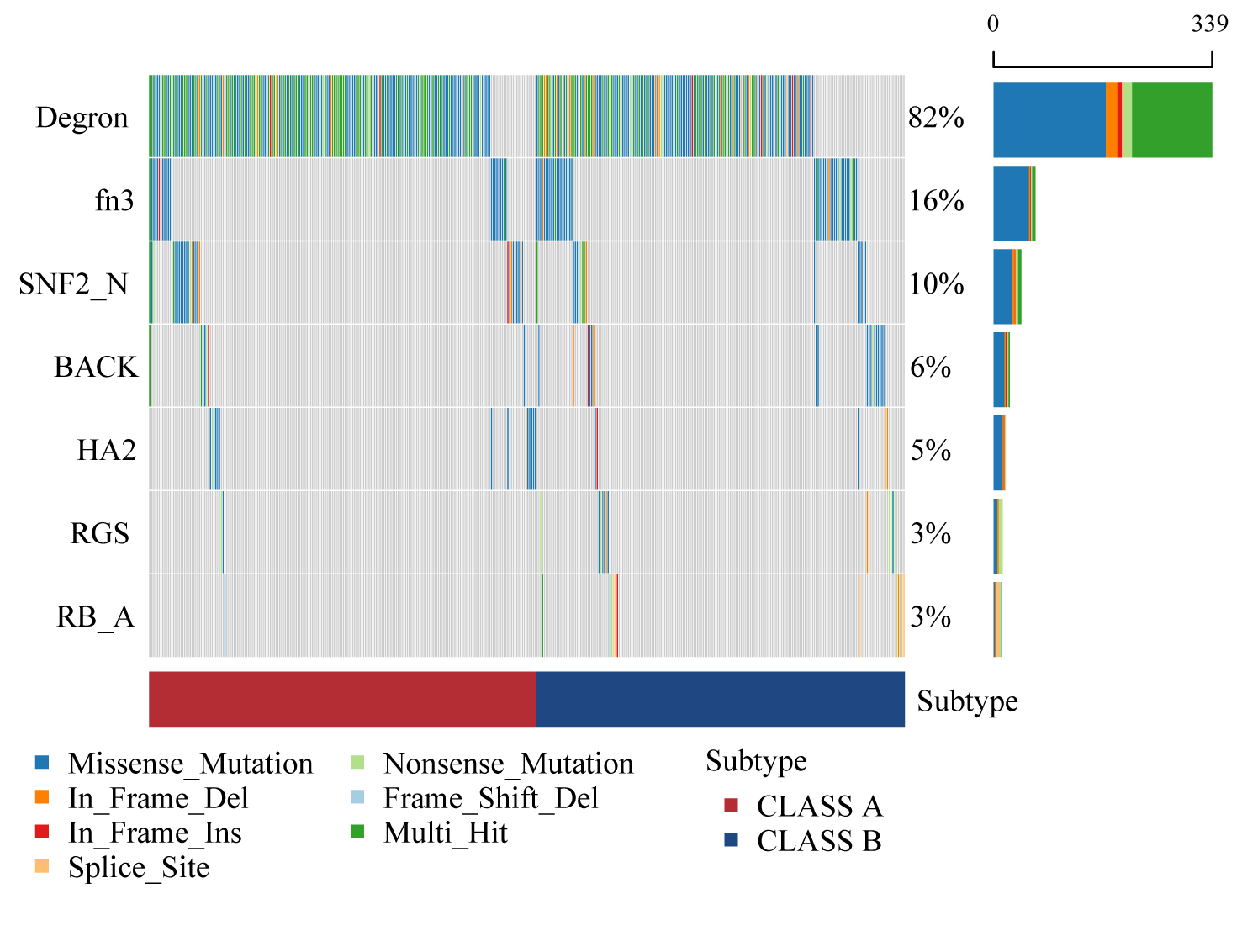

Supplement: S11 Fig — (TIF) [file pcbi.1012113.s011.tif]
